# CD8^+^ T cell depletion promotes human Tph/Tfh cell proliferation and Sjögren syndrome–like symptoms in PBMC-based humanized mice

**DOI:** 10.1172/jci.insight.191700

**Published:** 2025-11-24

**Authors:** Mariam Piruzyan, Sota Fujimori, Ryota Sato, Yuki Imura, Sachiko Mochiduki, Kana Takemoto, Akiko Nishidate, Yuzo Koda

**Affiliations:** Oncology & Immunology Unit, Research Division, Mitsubishi Tanabe Pharma Corporation, Yokohama, Kanagawa, Japan.

**Keywords:** Autoimmunity, Immunology, Inflammation, Autoimmune diseases, T cells

## Abstract

Peripheral helper T (Tph) and follicular helper T (Tfh) cells are key regulators of B cell differentiation and antibody production, making them promising targets for autoimmune disease treatment. However, their differentiation mechanisms differ significantly between humans and mice, limiting drug validation in mouse models. Here, we present a simple and effective method for in vivo proliferation of human Tph/Tfh and B cells. We discovered that after depleting CD8^+^ T cells of human peripheral blood mononuclear cell–transferred immunodeficient mice (CD8TΔhPBMC mice), human Tph/Tfh cells and B cells proliferated markedly in the spleen compared with those in human PBMC–transferred immunodeficient mice (hPBMC mice). Transcriptome analysis confirmed proliferating cells’ close resemblance to human Tph/Tfh cells. Furthermore, multicolor flow cytometry revealed CXCL13^+^ Tph cells infiltrating Sjögren’s syndrome–associated (SjS-associated) organs, such as salivary glands. Single-cell RNA sequencing identified IL-21^+^CXCL13^+^IFN-γ^+^ICOS^+^TIGIT^+^GPR56^+^ Tph cells in the salivary glands. These findings are consistent with reduced saliva volume and elevated SjS markers, such as anti-SSA antibody, in these mice, which were both ameliorated by immunosuppressants. In vitro, CD8^+^ T cells from hPBMC mice induced B cell apoptosis and inhibited Tph/Tfh differentiation. This model advances understanding of human Tph/Tfh cell biology and offers a valuable platform for studying SjS and therapeutic targets.

## Abstract 2

This study provides a simple and effective method for proliferation of human Tph/Tfh cells and induction of Sjögren syndrome-like symptoms in vivo using humanized mice.

## Graphical abstract

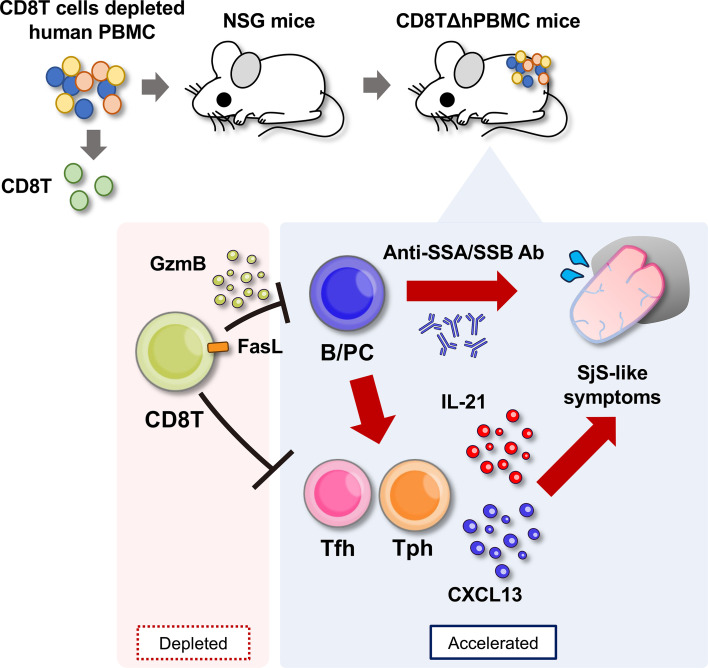

## Introduction

The discovery of follicular helper T (Tfh) and peripheral helper T (Tph) cells has revealed the mechanism of antibody production through the interaction between T and B cells and shed light on the pathogenesis of autoimmune diseases (1, 2). Tfh cells are a CD4^+^ T cell subset that expresses CXCR5 in addition to programmed cell death 1 (PD-1) and helps B cells in the interfollicular zone of secondary lymphoid organs, contributing to antibody production through B cell differentiation and maturation via IL-21, CD40L, and inducible costimulators (ICOS) (3, 4). Conversely, Tph cells are a CD4^+^ T cell subset discovered in 2017 and are characterized by their ability to induce local attraction of Tfh cells/B cells and ectopic lymphoid follicle formation through CXCL13 production in nonlymphoid tissues (5, 6). Tph cells function to help B cells through IL-21, CD40L, and ICOS similar to Tfh cells (2, 5, 7, 8). However, Tph cells do not express CXCR5 and are defined as CD4^+^PD-1^+^CXCR5^–^ cells. Instead, they express chemokine receptors, such as CCR2 and CX3CR1, involved in cell migration to inflammatory sites, and function in response to recruitment to inflammatory sites (9). Furthermore, Tph cells, although being CD4^+^ T cells, exhibit cytotoxicity through factors, such as IFN-γ, perforin, and granzyme, a major difference from Tfh cells (10). Many reports have emerged from the analysis of human samples indicating the involvement of Tph cells in the pathogenesis of immune diseases (8, 11–16).

In recent years, limitations to analyses using classical mouse models and in vitro systems using cell lines have become apparent, and the importance of directly understanding human pathophysiology through samples particularly from patients with diseases is increasing. Among them, the differentiation mechanisms and functional mechanisms of Tph/Tfh cells have been known to differ significantly between humans and mice. Human Tph/Tfh cells can be induced by soluble factors, such as IL-12 and TGF-β1/3 (17, 18). Conversely, TGF-β1 acts as an inhibitory factor in mice (19). Mouse Tfh cells require coexistence with germinal center B cells for their differentiation and maintenance (20, 21). Additionally, T cells and follicular dendritic cells (FDCs), crucial for Tfh and CXCR5^+^ B cell attraction, are considered the main sources of CXCL13 in humans, whereas FDCs and stromal cells are considered to perform these functions in mice (22–27). Hence, there are considerable limitations to utilizing findings from mouse models in human pathophysiology and studying functions and differentiation mechanisms in diseases; thus, a model that induces human Tph and Tfh cells in vivo should be developed. However, no current in vivo mouse model has resolved this challenge.

Sjögren’s syndrome (SjS) is an autoimmune disease characterized by lymphocyte infiltration mainly of T and B cells into the salivary and lacrimal glands, resulting in glandular dysfunction and dryness (28). In severe cases, the pathophysiology spreads to extraglandular tissues, such as the lung and pancreas, resulting in lymphomas (28). Although biological agents, such as rituximab and abatacept, are being tested, no disease-modifying drugs have been approved for this disease (29–31). SjS is characterized by the ectopic formation of lymphoid follicles and the involvement of Tph cells, which are central to ectopic lymphoid follicle formation. Recent reports investigating patients with SjS also suggest Tph cell involvement in the pathophysiology (32–34). Nonobese diabetic (NOD) mice have been known as a mouse model for SjS, as T cells spontaneously infiltrate the salivary and lacrimal glands similar to the pancreatic symptoms seen in type I diabetes (35, 36). However, this model has limitations, as it rarely exhibits a decrease in saliva production, and the cells involved in SjS pathogenesis, such as Tph cells, are markedly different from those in humans.

In this study, a humanized mouse model generated by transferring CD8^+^ T cell–depleted peripheral blood mononuclear cells (PBMCs) into immunodeficient mice was used to efficiently expand human Tph/Tfh cells in vivo and establish a potentially novel disease model that induces SjS-like symptoms through Tph cells. Originally, to investigate the effects of CD8^+^ T cells in humanized PBMC (hPBMC) mice, PBMCs were transferred into immunodeficient mice depleted of CD8^+^ T cells (37), and incidentally, we discovered that Tph/Tfh cells proliferate efficiently in CD8TΔhPBMC mice. Even in the lack of CD8^+^ T cell–dependent graft-versus-host disease (GVHD) symptoms seen in hPBMC mice, highly activated Tph cells expressing CXCL13, IL-21, and IFN-γ infiltrated the salivary glands and induced severe reduction in saliva production. This study provides a tool for human Tph/Tfh cell studies and a mouse disease model that overcomes the limitations of classical SjS models.

## Results

### CD8^+^ T cell depletion promotes human Tph, Tfh, and B cell proliferation in hPBMC mice.

**Figure 1:**
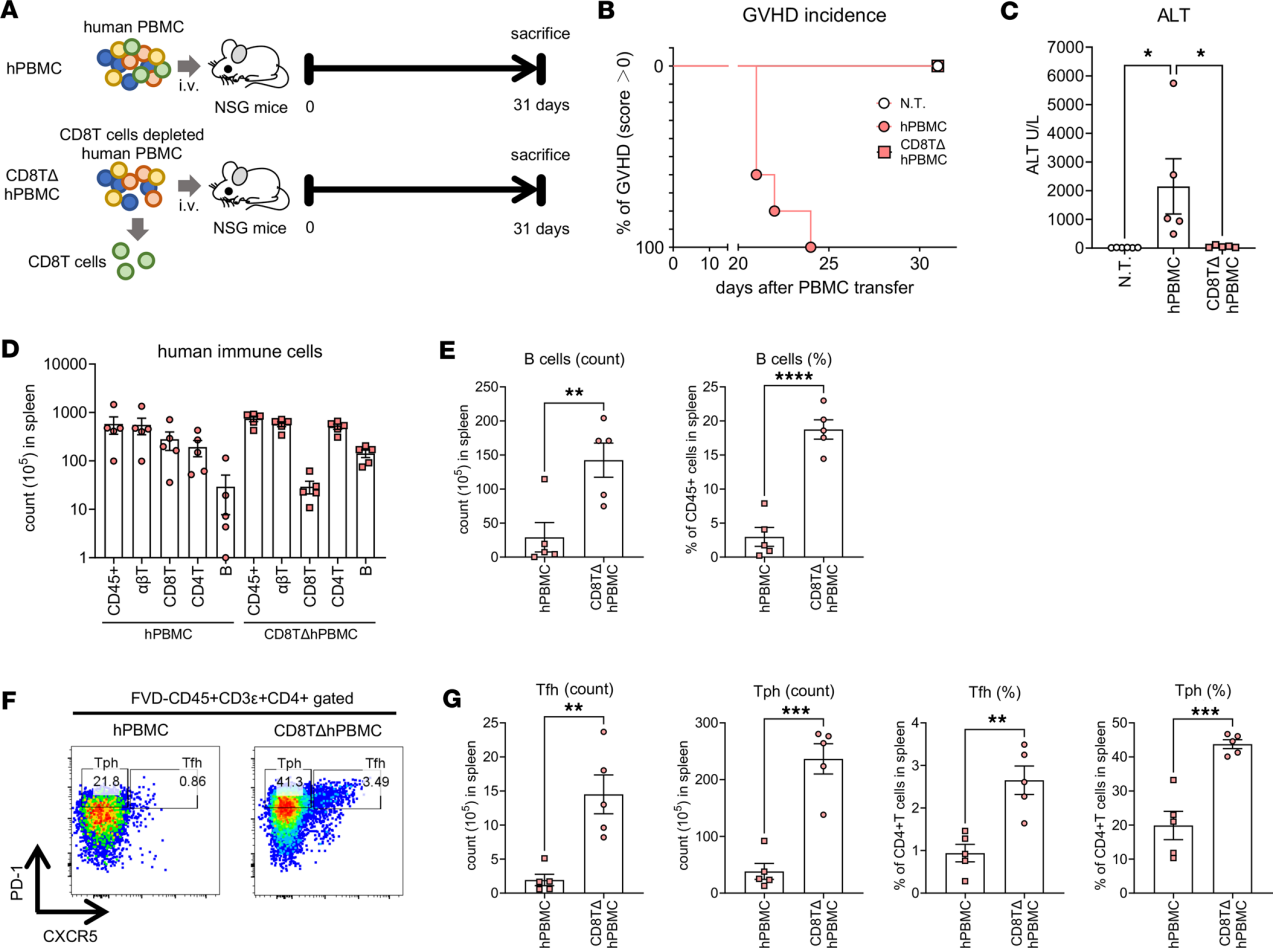
CD8^+^ T cell depletion enhances Tph, Tfh, and B cell proliferation in hPBMC mice. NSG mice were intravenously injected with human PBMCs or CD8^+^ T cell–depleted PBMCs. Mice were sacrificed when GVHD-like symptoms were observed or on day 31 after injection of PBMCs. (**A**) Study design showing construction of CD8^+^ T cell–depleted PBMC-transferred humanized mice. (**B**) GVHD score change (*n* = 5 or 6 per group). (**C**) ALT activity in the serum (*n* = 5 or 6 per group). (**D**) The number of human immune cells in the spleen (*n* = 5 per group). (**E**) The number of B cells and B cell percentage of CD45^+^ cells in the spleen (*n* = 5 per group). (**F**) Gating strategy of Tph and Tfh cells. (**G**) The number of Tfh and Tph cells and Tfh and Tph percentage of CD4^+^ T cells in the spleen. Data are presented as means with SEM. *P < 0.05, **P < 0.01, ***P < 0.001, ****P < 0.0001; statistical analysis was performed using 1-way ANOVA with Tukey’s multiple-comparison post hoc test (**C**) or 2-tailed Student’s *t* test (**E** and **G**). Data are representative of more than 3 independent experiments. ALT, alanine aminotransferase; NSG, NOD/SCID IL-2 receptor γ^–/–^; N.T., nontreatment.

hPBMC mice with an immune system primarily composed of T cells can be created by transplanting human PBMCs into immunodeficient mice. In this study, we examined the role of CD8^+^ T cells in GVHD and the composition of human immune cells in these mice by inducing the lack of CD8^+^ T cells in mice with transplanted PBMCs (CD8TΔhPBMC mice) and observed their phenotypes (Figure 1A and Supplemental Table 1; supplemental material available online with this article; https://doi.org/10.1172/jci.insight.191700DS1). CD8^+^ T cells were depleted by magnetic beads from PBMCs, and we confirmed the depletion by flow cytometry (Supplemental Figure 1). Consistent with previous reports (37, 38), the removal of CD8^+^ T cells markedly improved GVHD symptoms and liver damage in hPBMC mice, indicating that these symptoms in hPBMC mice are CD8^+^ T cell dependent (Figure 1, B and C). Surprisingly, despite the absence of GVHD symptoms, B and CD4^+^ T cells were significantly increased in CD8TΔhPBMC mice (Figure 1, D and E). The majority of cells in the B cell lineage were plasmablasts and plasma cells, indicating induced B cell differentiation and activation in CD8TΔhPBMC mice (Supplemental Figure 2). This suggests the promotion of antibody production by B cells. Among the increased CD4^+^ T cell subsets, the proportions of CXCR5^+^PD-1^+^ Tfh cells and CXCR5^–^PD-1^+^ Tph cells were markedly elevated, exhibiting a skewed composition toward these subsets (Figure 1, F and G). Furthermore, high reproducibility was observed in B, Tfh, and Tph cell proliferation in other healthy donors with different backgrounds and sex compared with CD8TΔhPBMC mice (Supplemental Figure 3 and Supplemental Table 2). Supplemental Figure 4 shows the profiles of other non-T and non-B cells. In this model, myeloid cells did not show significant engraftment. Because exogenous growth factors, such as human granulocyte-macrophage colony-stimulating factor (GM-CSF) and IL-3, are necessary for myeloid cell engraftment in hPBMC mice (39), this likely applies to CD8TΔhPBMC mice. These results indicate that CD8TΔhPBMC mice can efficiently induce human-specific differentiation mechanisms of Tph and Tfh cells in vivo without accompanying GVHD symptoms.

### Characteristic genetic signatures of Tph and Tfh cells in spleens of CD8TΔhPBMC mice.

**Figure 2:**
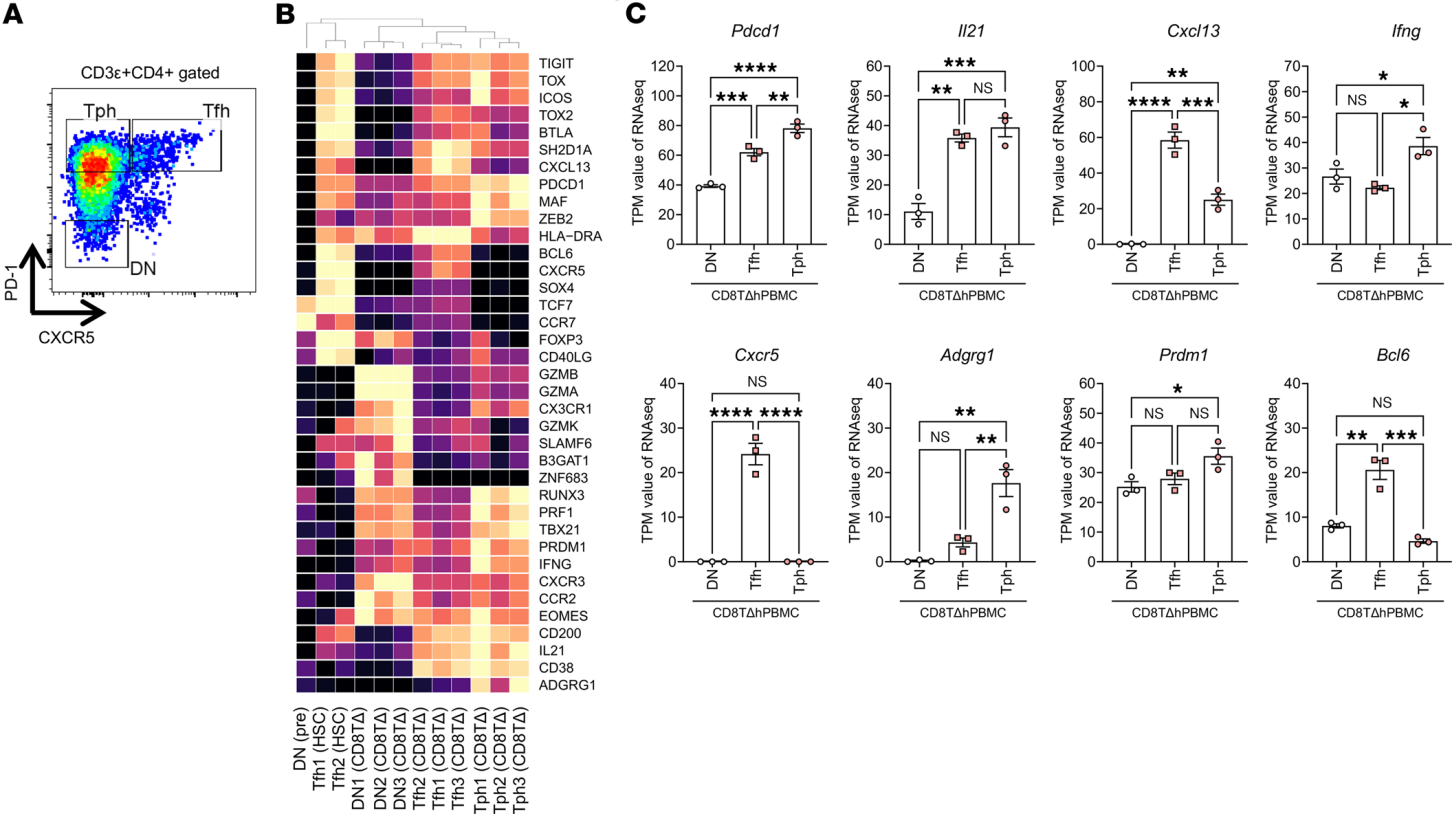
Gene expression patterns of Tph and Tfh cells proliferate in the spleens of CD8TΔPBMC mice. Gene expression profiling of the spleen tissue from HSC and CD8TΔhPBMC mice was performed using RNA-Seq analysis (*n* = 3 per group). (**A**) Gating strategy of Tph and Tfh cells. (**B**) Heatmap showing characteristic genes in the spleens of HSC and CD8TΔhPBMC mice. (**C**) PDCD1, IL21, CXCL13, IFNG, CXCR5, ADGRG1, PRDM1, and BCL6 gene expression in the spleen. Data are presented as means with SEM. *P < 0.05, **P < 0.01, ***P < 0.001, ****P < 0.0001; significantly different (1-way ANOVA with Tukey’s multiple-comparison post hoc test). Data are combined from 2 experiments. DN, PD-1 and CXCR5 double-negative cells; TPM, transcripts per million; HSC, hematopoietic stem cell; ADGRG1, adhesion G protein-coupled receptor G1; PRDM1, PR/SET domain 1; BCL6, B-cell CLL/lymphoma 6.

We comprehensively analyzed the gene expression patterns of human Tph/Tfh cells derived from CD8TΔhPBMC mice and compared them with the known gene expression profiles of Tph/Tfh cells. These cells were isolated from the spleens of CD8TΔhPBMC mice using FACS and analyzed via RNA-Seq. The gating strategy for sorting is shown in Figure 2A. The target cells were confirmed to be isolated with high specificity, and gene detection was found to be successful based on PD-1 and CXCR5 expression patterns (Figure 2, B and C). Human Tph/Tfh cells from CD8TΔhPBMC mice markedly expressed IL-21 and CXCL13, the defining markers of these cells, compared with those in double-negative cells. Notably, Tph/Tfh cells from CD8TΔhPBMC mice exhibited higher IL-21 expression than in Tfh cells from HSC-transplanted humanized mice, indicating a highly activated state. Furthermore, Tph cells expressed transcription factor markers PRDM1 (encoding BLIMP-1) and MAF, whereas Tfh cells expressed BCL6, reflecting typical Tph/Tfh cell characteristics. Cytotoxic factors such as IFN-γ and perforin, characteristics of Tph cells, were also expressed in Tph cells from CD8TΔhPBMC mice. Moreover, ADGRG1 (GPR56), known to be highly expressed in Tph cells of patients with anti-citrullinated protein antibody–positive rheumatoid arthritis (40), was prominently expressed in cells from CD8TΔhPBMC mice. These findings reveal that Tph/Tfh cells from CD8TΔhPBMC mice closely mimic typical Tph/Tfh cells, demonstrating the potential use of CD8TΔhPBMC mice as a tool for studying the differentiation mechanisms and pathological roles of these cells.

### CXCL13^+^IL-21^+^ Tph cell infiltration in salivary glands of CD8TΔPBMC mice.

**Figure 3:**
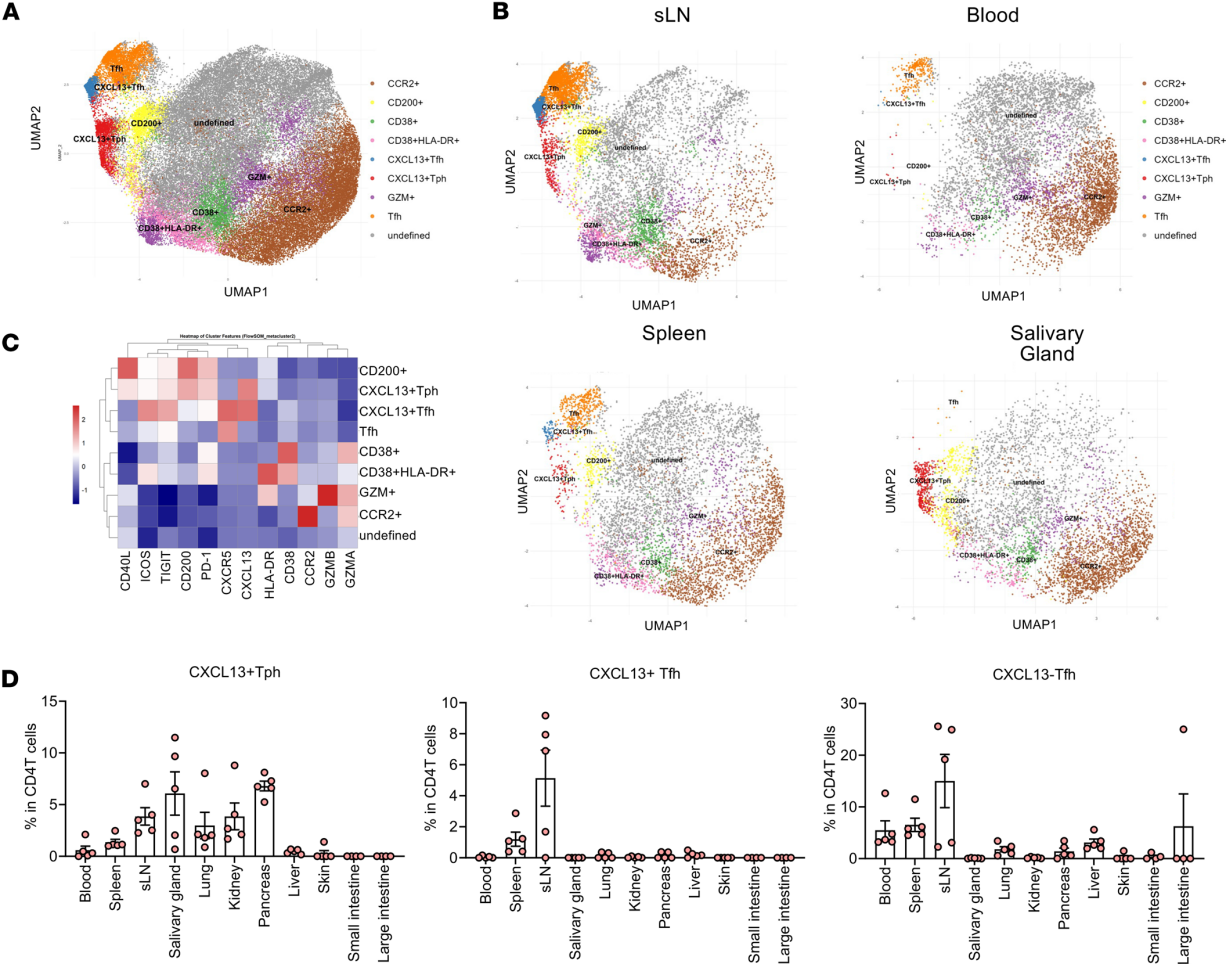
CXCL13^+^ Tph cell infiltration in various organs of CD8TΔPBMC mice. (**A**) Uniform manifold approximation and projection (UMAP) plot showing CD4^+^ T cell clusters of all samples. (**B**) UMAP dimensional reduction and FlowSOM metacluster of the submandibular lymph node (sLN), blood, spleen, and salivary gland–infiltrating CD4^+^ T cells. (**C**) Representative expression of the surface and intracellular markers of CD4^+^ T cells. (**D**) CXCL13^+^ Tph, CXCL13^+^ Tfh, and CXCL13^–^ Tfh cell percentage in CD4^+^ T cells in various organs of CD8TΔPBMC mice (*n* = 5 per group). Data are presented as means with SEM. Data are representative of 2 independent experiments.

**Figure 4:**
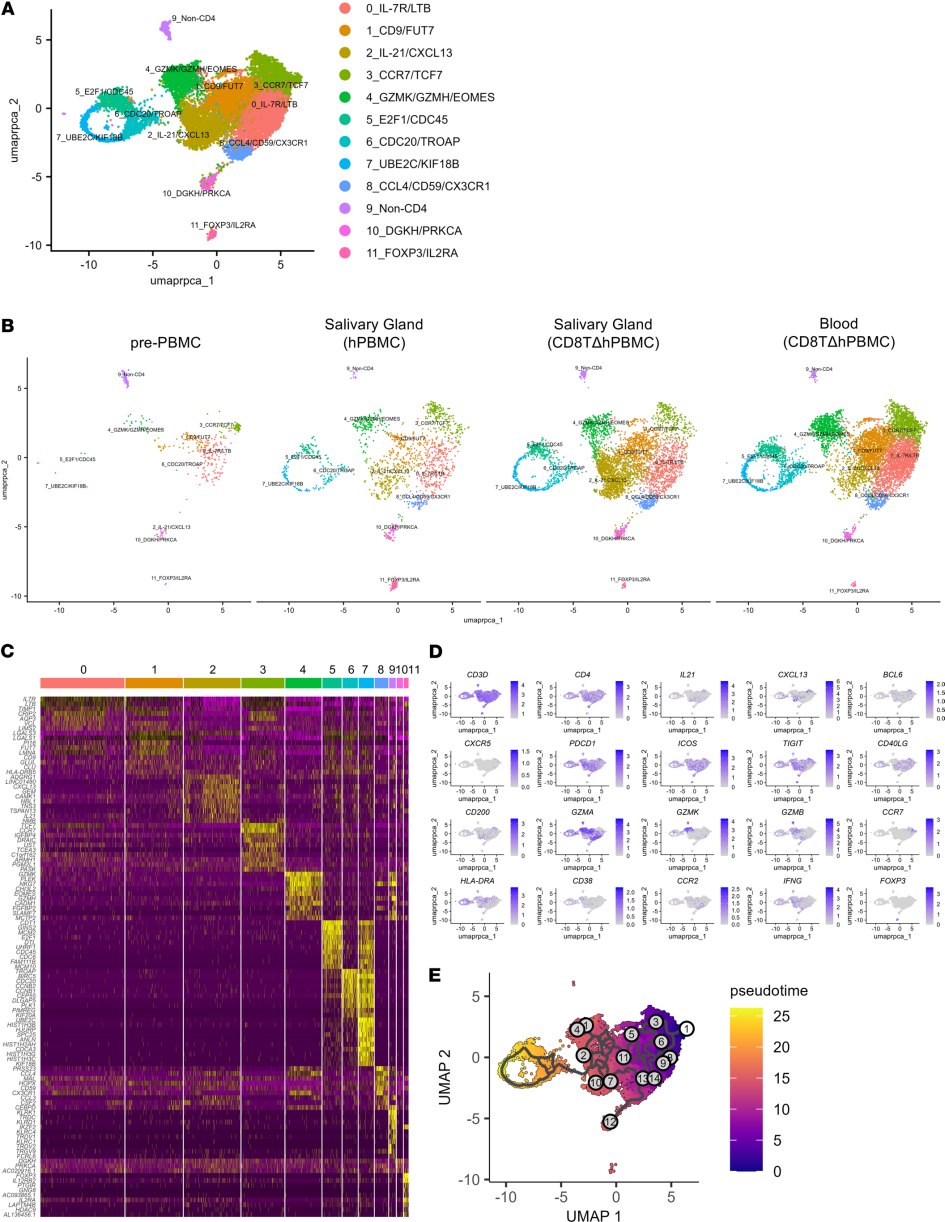
CXCL13^+^IL-21^+^ Tph cell infiltration in salivary glands of CD8TΔPBMC mice. Single-cell RNA sequencing was performed using CD4^+^ T cells from pretransfer PBMCs (pre-PBMC, *n* = 426), salivary glands of human PBMC–transferred mice (PBMC mice, *n* = 1,575), salivary glands of CD8^+^ T cell–depleted human PBMC-transferred mice (CD8TΔPBMC mice, *n* = 4,446), and blood of CD8TΔPBMC mice (*n* = 7,131). (**A**) UMAP plot showing CD4^+^ T cell clusters of all samples and (**B**) each sample. (**C**) A gene expression heatmap showing the top 10 differentially expressed genes for each cluster. (**D**) Feature plots showing expression of indicated genes in CD4^+^ T cells. (**E**) Pseudotime trajectory inferred using Monocle3. Cells are colored based on their pseudotime values, indicating the progression of cellular differentiation. Samples from each group were pooled from 4 mice. Data are representative of a single experiment.

To further characterize these Tph/Tfh cells and their systemic localization, we performed comprehensive marker expression analysis for CD4^+^ T cells in various organs using spectral flow cytometry. The gating strategy for flow cytometry is shown in Supplemental Figure 5. Clustering analysis based on 12 markers classified CD4^+^ T cells into 9 clusters (CCR2^+^, CD200^+^, CD38^+^, CD38^+^HLA-DR^+^, CXCL13^+^ Tfh, CXCL13^+^ Tph, Tfh [CXCL13^–^ Tfh], granzyme-positive [GZM^+^], and undefined) (Figure 3A and Supplemental Figure 6). Interestingly, Tfh cells primarily infiltrated the spleen and lymph nodes, and CXCL13^+^ Tph cells infiltrated the spleen and SjS-related organs, such as the salivary glands, pancreas, kidneys, and lungs (Figure 3, B–D, and Supplemental Figure 7). In the blood, CCR2^+^CD4^+^ T cells were abundant, indicating that these blood cells might infiltrate tissues and differentiate into CXCL13^+^ Tph cells. To further assess the characteristics of Tph cells infiltrating the salivary glands, we performed single-cell RNA-Seq (scRNA-Seq) analysis of CD4^+^ T cells from the salivary glands. Cluster analysis revealed, similar to flow cytometry results, a significant greater number of CXCL13^+^ Tph cell clusters in the salivary glands of CD8TΔhPBMC mice compared with in hPBMC mice (Figure 4, A–D). These clusters selectively expressed IL21, ADGRG1, and Tph-related molecules, such as ICOS, CD40LG, and TIGIT (Figure 4, C and D). Velocity analysis indicated that these clusters were extensively differentiated versus clusters 3, 0, and 1, which included naive or immature cells, demonstrating the presence of activated effector cells in the salivary glands (Figure 4E). These findings suggest that Tph cells infiltrating the salivary glands in CD8TΔhPBMC mice may pathologically affect salivary gland function, contributing to human disease pathology.

### CD8TΔPBMC mice exhibit SjS-like symptoms and tacrolimus administration ameliorates these symptoms.

**Figure 5:**
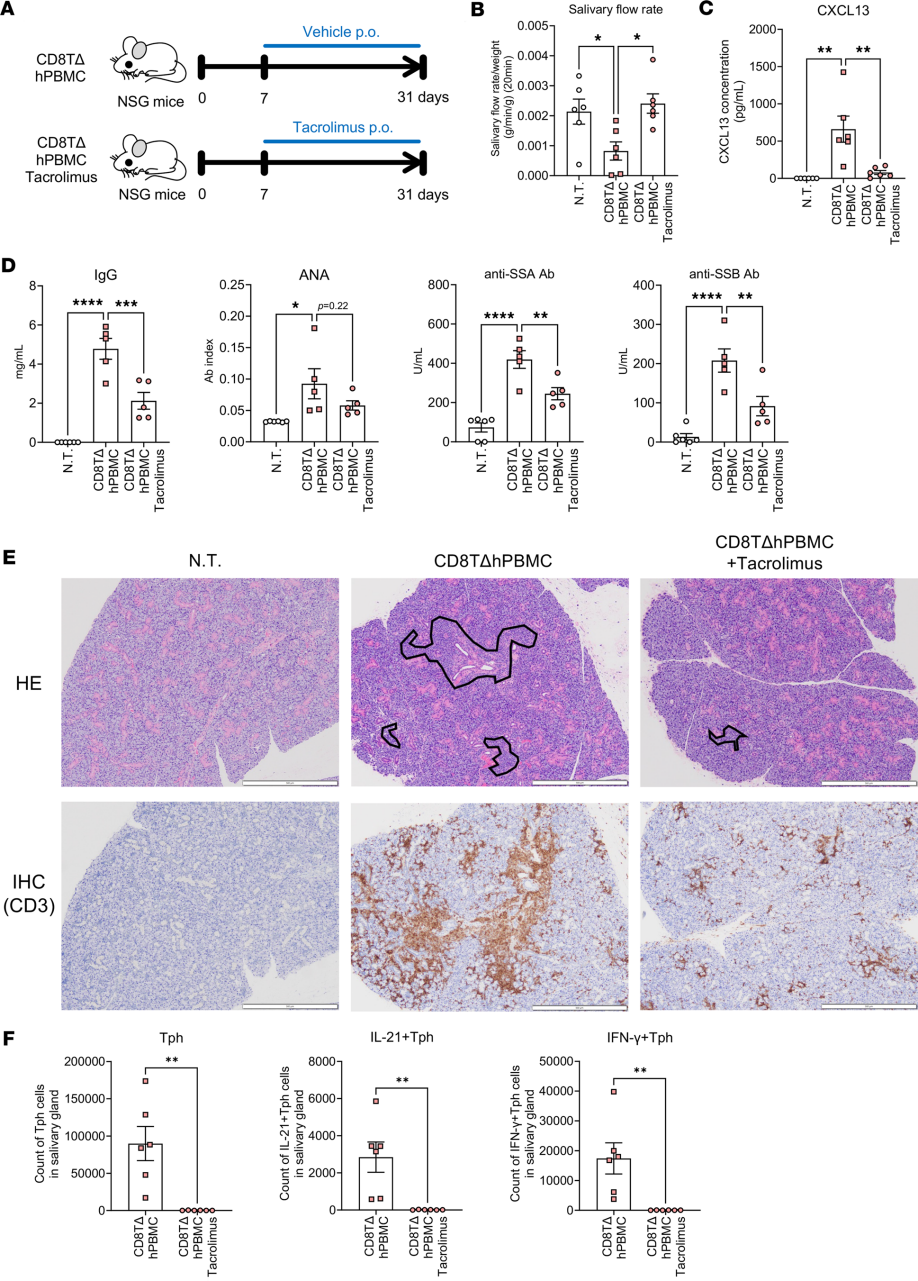
CD8TΔPBMC mice show SjS-like symptoms in Tph cell–dependent manner. NSG mice were intravenously injected with CD8^+^ T cell–depleted PBMCs and treated daily with oral tacrolimus (*n* = 6 per group). Mice were sacrificed on day 31 (**A**–**E**). (**A**) Study design. (**B**) The salivary flow rate was measured on day 28 (*n* = 6). (**C**) CXCL13 concentration in plasma (*n* = 6). (**D**) Plasma concentrations of IgG, ANA, anti-SSA antibody (Ab), and anti-SSB Ab. (**E**) Representative photomicrographs of HE-stained and IHC- (CD3) stained salivary gland sections. Black sections show the area of infiltrated immune cells. Scale bars, 500 μm. NT, nontreated mice; hPBMCΔCD8T, CD8^+^ T cell–depleted human PBMC-transferred mice; HE, hematoxylin and eosin; IHC, immunohistochemistry. (**F**) The number of human immune cells in salivary glands (*n* = 6 per group). Data are presented as means with SEM. *P < 0.05, **P < 0.01, ***P < 0.001, ****P < 0.0001. Statistical analysis was performed using 1-way ANOVA with Tukey’s multiple-comparison post hoc test (**B**–**D**) or Student’s *t* test (**F**). Data are representative of more than 3 independent experiments.

**Figure 6:**
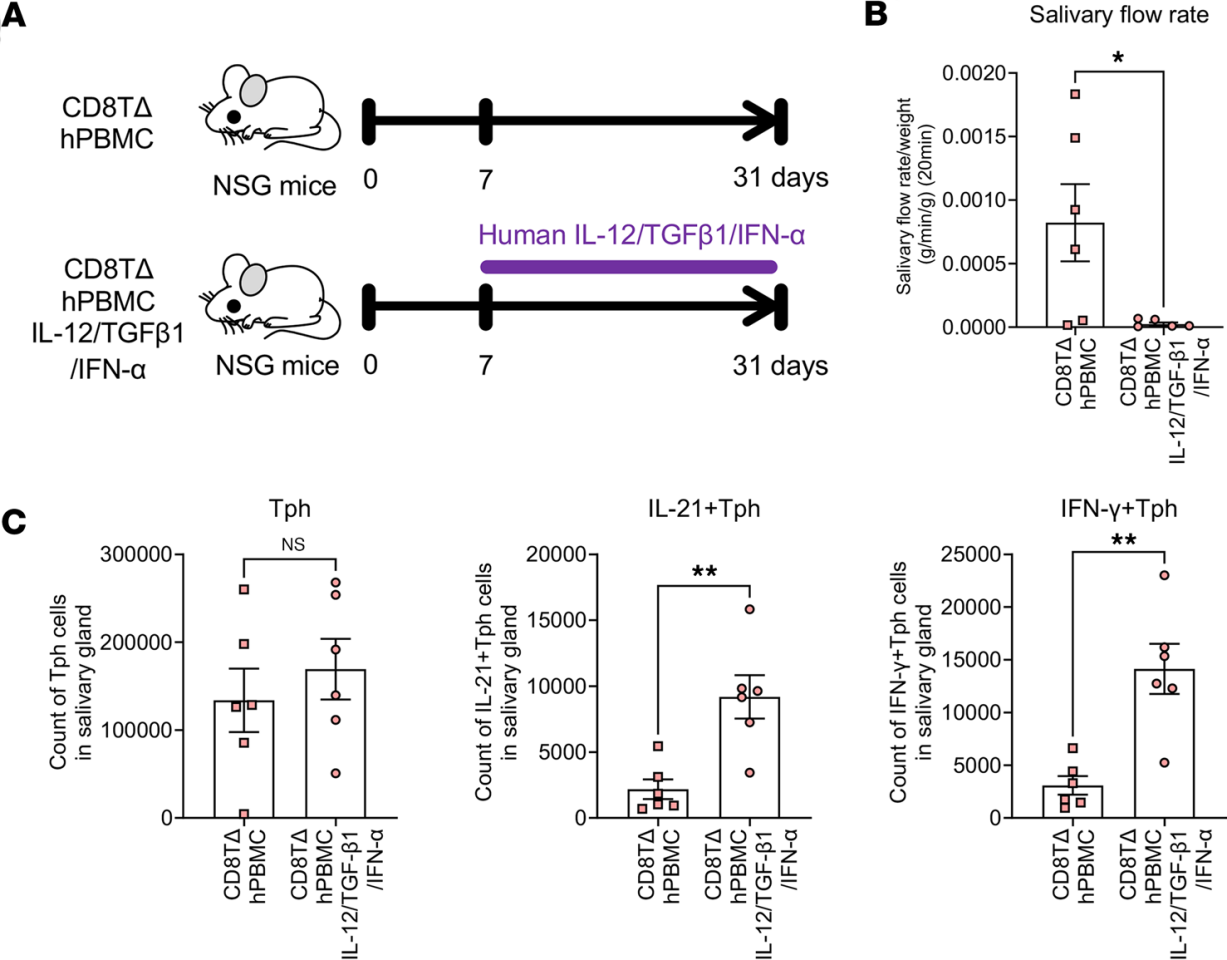
The salivary gland symptoms of CD8TΔhPBMC mice are exacerbated by the Tph cell–inducing factors. NSG mice were intravenously injected with CD8^+^ T cell–depleted PBMCs and subcutaneously injected with human IL-12, TGF-β1, and IFN-α 3 times per week starting from day 7 (*n* = 5 or 6 per group). Mice were sacrificed on day 30 postinjection. (**A**) The study design of cytokine injection. (**B**) The salivary flow rate was measured on day 28 (*n* = 5 or 6 per group). (**C**) The number of human immune cells in salivary glands (*n* = 6 per group). Data are presented as means with SEM. *P < 0.05, **P < 0.01. Statistical analysis was performed using Student’s *t* test (**B** and **C**). Data are representative of 2 independent experiments.

**Figure 7:**
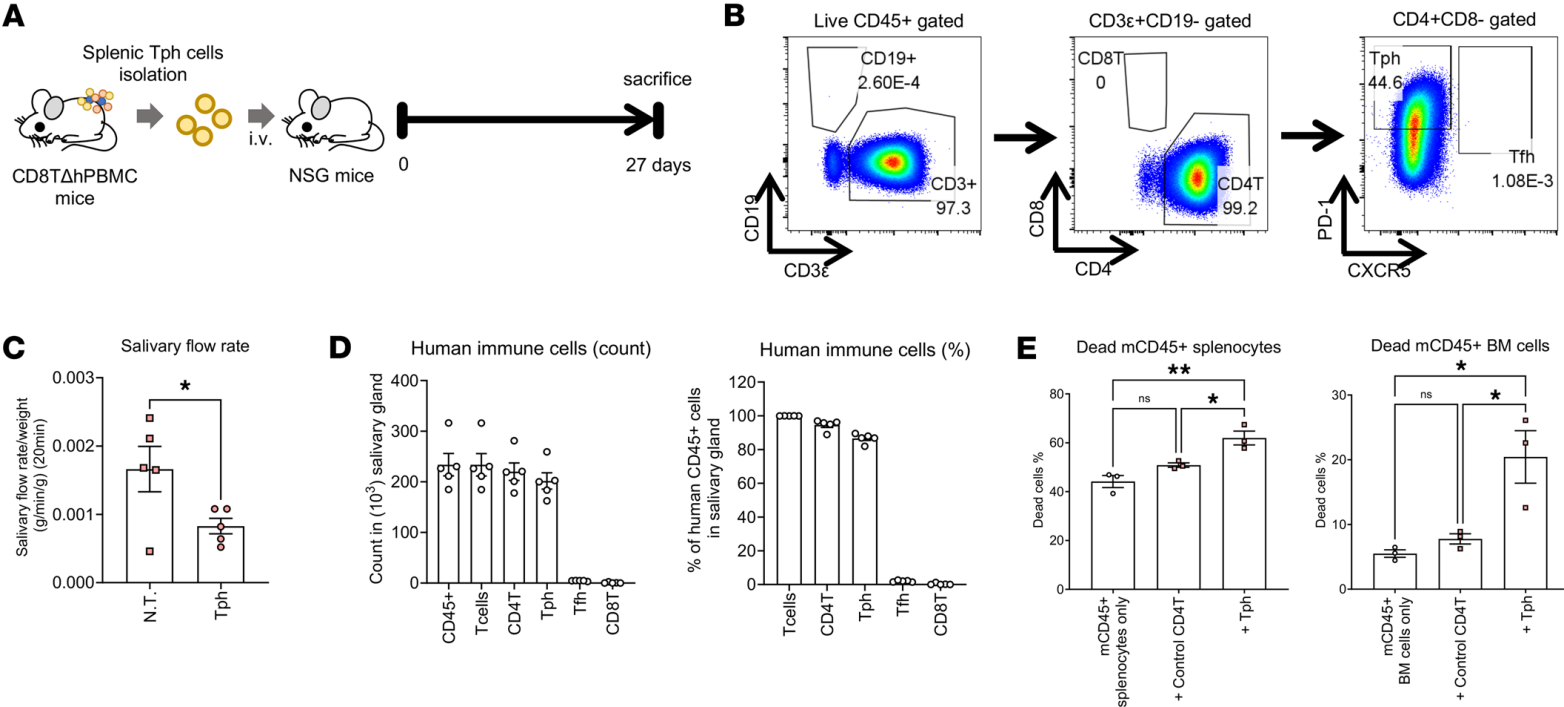
The transfer of Tph cells from CD8TΔhPBMC mice induces salivary gland symptoms in immunodeficient mice. Tph cells were isolated from the spleens of CD8TΔhPBMC mice. NSG mice were intravenously injected with isolated Tph cells (*n* = 5 per group). Mice were sacrificed on day 27. (**A**) Study design. (**B**) Representative CD19/CD3ε, CD8/CD4, PD-1/CXCR5 staining of transferred Tph cells. (**C**) The salivary flow rate was measured on day 26. (**D**) The numbers and percentages of human immune cells in the salivary gland. (**E**) Tph cells isolated from the spleens of CD8TΔhPBMC mice were cultured with mouse CD45^+^ splenocytes or mouse CD45^+^ bone marrow (BM) cells for 16 hours (*n* = 3). The percentage of dead cells is shown. Data are presented as means with SEM. *P < 0.05, **P < 0.01. Statistical analysis was performed using Student’s *t* test (**C**) or 1-way ANOVA with Tukey’s multiple-comparison post hoc test (**E**). Data are combined from 2 experiments.

Considering the marked infiltration of activated Tph cells into the salivary glands, the salivary gland function of CD8TΔhPBMC mice and the effects of drug intervention were examined (Figure 5A). As expected, decreased saliva production and increased lymphocyte infiltration in the salivary and lacrimal glands and plasma CXCL13 increase were observed in CD8TΔhPBMC mice as seen in SjS (Figure 5, B and C, and Supplemental Figure 8). Furthermore, a significant increase in plasma IgG, anti-nuclear antibody (ANA), and anti-SSA and anti-SSB antibodies, which are disease-related markers of SjS, was observed in CD8TΔhPBMC mice (Figure 5D). Additionally, marked lymphocytic infiltration was confirmed by hematoxylin and eosin staining and immunostaining of human T cells in CD8TΔhPBMC mice (Figure 5E). These SjS-like symptoms were markedly ameliorated by the administration of tacrolimus, an immunosuppressant targeting the T cells (Figure 5, B–E). Tacrolimus treatment markedly reduced IL-21– and IFN-γ–producing Tph cells in the salivary glands, revealing that these SjS-like symptoms are caused by activated Tph cells (Figure 5F). Furthermore, in vivo, the administration of cytokines known to activate Tph cells (IL-12, TGF-β1, and IFN-α) further reduced saliva production and worsened SjS-like symptoms in CD8TΔhPBMC mice (Figure 6, A and B). Although the number of Tph cells infiltrating the salivary glands did not change upon cytokine administration, the number of IL-21– and IFN-γ–producing activated Tph cells significantly increased, indicating that Tph cell activation exacerbates salivary gland symptoms (Figure 6C). To further investigate Tph cell pathogenicity, we isolated Tph cells that were increased in the spleens of CD8TΔhPBMC mice and retransplanted them into immunodeficient mice (Figure 7A). Assessment of the purity of the Tph cells before transfer verified successful isolation (Figure 7B). The results of retransplantation confirmed that the transferred Tph cells infiltrated the salivary glands, and saliva volume was significantly decreased, even with the transfer of Tph cells alone (Figure 7, C and D). Additionally, in vitro coculture of these Tph cells with spleen and bone marrow cells from recipient mice confirmed that Tph cells exhibit cytotoxicity (Figure 7E). These observations indicate that CD8TΔhPBMC mice represent a potentially novel disease model exhibiting Tph-dependent SjS-like symptoms. Furthermore, in vivo and in vitro experiments demonstrated that these Tph cells possess aspects of CD4^+^ cytotoxic lymphocytes (CTLs).

Additionally, because Tph and Tfh cell infiltration was observed in organs expressing extraglandular SjS symptoms, we investigated the pathological features and biomarker changes in each tissue for the presence of extraglandular symptoms in this model (Supplemental Figures 8 and 9 and Supplemental Tables 3 and 4). Examination of markers of pancreatic dysfunction, such as blood glucose and urinary glucose levels, did not show a definitive increase (Supplemental Figure 9 and Supplemental Table 4). However, a significant increase in surfactant protein D (SP-D), a marker of lung dysfunction, was observed in the lungs, and renal tissue damage was observed in the kidneys upon histopathological examination (Supplemental Figures 8 and 9 and Supplemental Table 3). Therefore, although no symptoms were observed in the pancreas, changes related to organ dysfunction were noted in organs expressing extraglandular symptoms of SjS, such as the lungs and kidneys, indicating the usefulness of this model for evaluating extraglandular symptoms.

### CD8^+^ T cells directly induce B cell apoptosis and inhibit Tph/Tfh cell differentiation in vitro.

**Figure 8:**
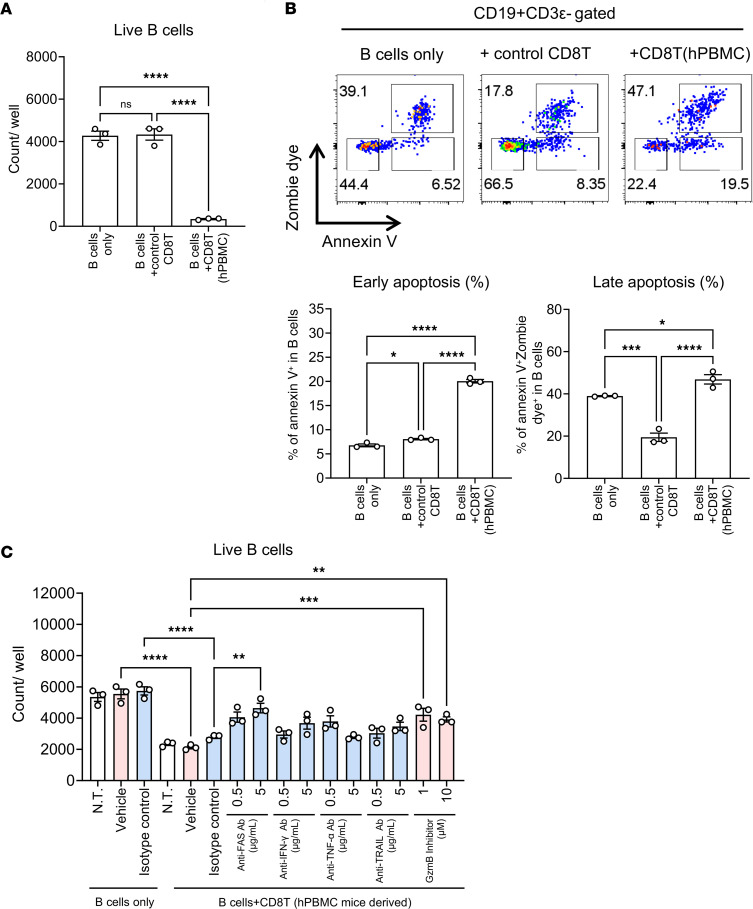
CD8^+^ T cells of hPBMC mice directly induce B cell apoptosis. CD8^+^ T cells were isolated from hPBMC-transferred NSG mice. CD8^+^ T cells were cultured with B cells (**A**–**C**). (**A**) The number of live B cells. (**B**) Representative annexin V/Zombie dye staining of cultured B cells and the percentage of early/late apoptotic B cells. (**C**) The effects of neutralizing antibodies (0.5 or 5 μg/mL) or inhibitors (1 or 10 μM) on B cells’ death were evaluated. The number of live B cells is shown. Data are presented as means with SEM (*n* = 3). *P < 0.05, **P < 0.01, ***P < 0.001, ****P < 0.0001; significantly different (1-way ANOVA with Tukey’s multiple-comparison post hoc test). Data are representative of 2 independent experiments.

To evaluate the mechanism by which CD8^+^ T cell depletion promotes B and Tph/Tfh cell proliferation, CD8^+^ T cells were derived from spleens of hPBMC mice and cocultured with PBMC-derived B or CD4^+^ T cells under CD3/CD28 stimulation. This resulted in significant reduction in the number of B cells (Figure 8A). Staining with apoptosis markers annexin V and amine-reactive fluorescent dye revealed a marked increase in early apoptosis (annexin V^+^Zombie dye^–^) and late apoptosis (annexin V^+^Zombie dye^+^) cells, indicating that hPBMC mouse–derived CD8^+^ T cells induce both early and late apoptosis in B cells (Figure 8B). Furthermore, the inhibition assay targeting molecules associated with CD8^+^ T cell effector function, such as granzyme B and perforin, was performed in cocultures of CD8^+^ T cells and B cells. The results showed that the phenotype was partially canceled by the inhibition of granzyme B or Fas/FasL, indicating that these factors contribute to the control of B cells by CD8^+^ T cells (Figure 8C). In contrast, coculturing hPBMC mouse–derived CD8^+^ T cells with CD4^+^ T cells did not affect CD4^+^ T cell numbers and further reduced CD4^+^ T cell apoptosis, suggesting that CD8^+^ T cells do not induce apoptosis in CD4^+^ T cells (including Tph/Tfh cells) (Supplemental Figure 10, A and B). However, CD8^+^ T cells significantly inhibited Tph/Tfh cell differentiation and proliferation under CD3/CD28 stimulation, without affecting other CD4^+^ T cell subsets, indicating that hPBMC mouse–derived CD8^+^ T cells specifically inhibit Tph/Tfh cell differentiation and proliferation through a nonapoptotic pathway (Supplemental Figure 10, C and D).

### B cell depletion decreases Tph/Tfh cells, IgG, anti-SSA antibody, and ANA in CD8TΔhPBMC mice.

**Figure 9:**
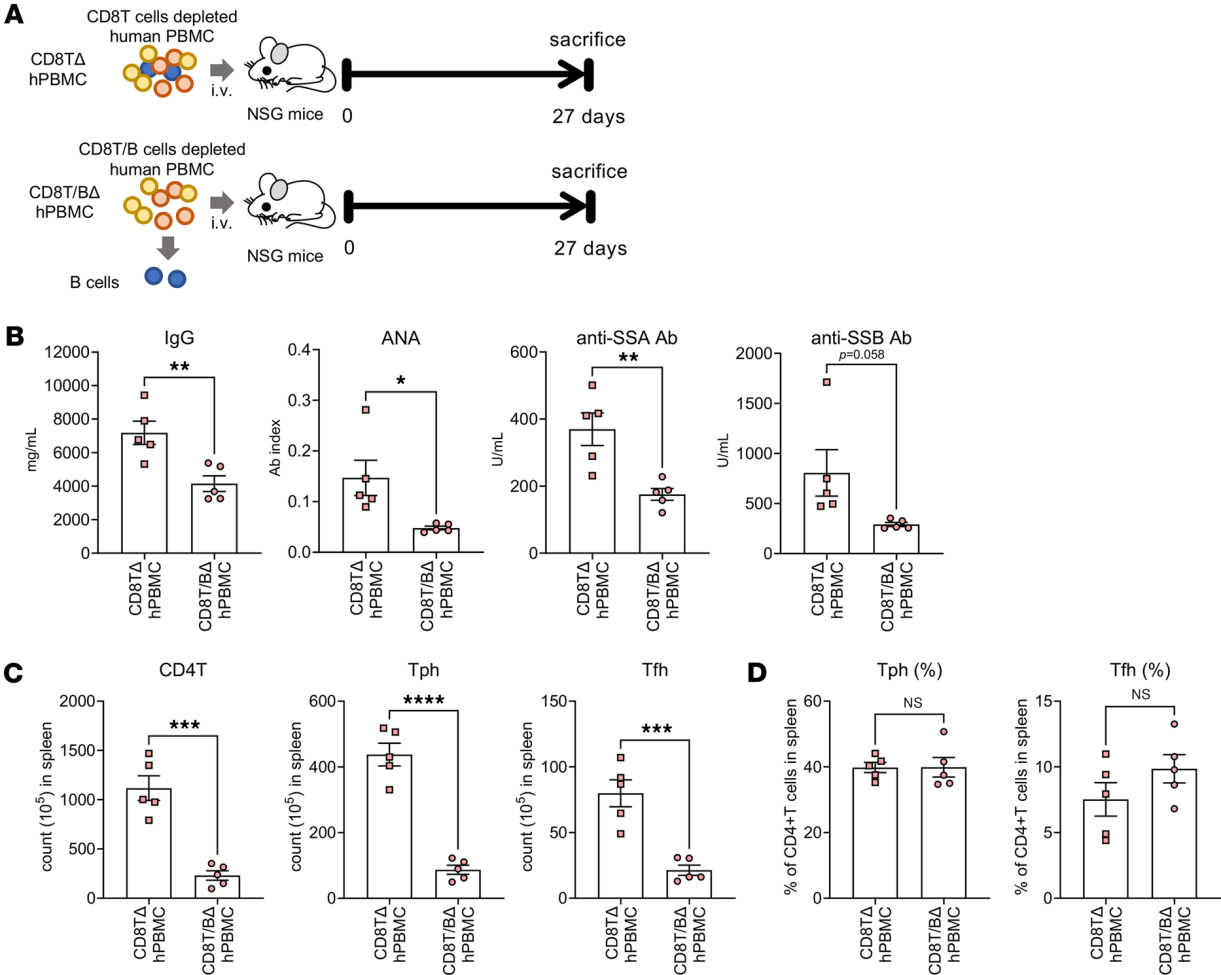
B cell depletion decreases Tph/Tfh cells, IgG, ANA, and anti-SSA antibody in CD8TΔhPBMC mice. NSG mice were intravenously injected with CD8^+^ T cell–depleted PBMCs or CD8^+^ T and B cell–depleted PBMCs. Mice were sacrificed on day 27 postinjection. (**A**) Study design. (**B**) Plasma concentrations of IgG, ANA, anti-SSA Ab, and anti-SSB Ab. (**C**) The number of CD4^+^ T, Tph, and Tfh cells in the spleen. (**D**) The percentage of Tph and Tfh cells among CD4^+^ T cells in the spleen. Data are presented as means with SEM (*n* = 5). *P < 0.05, **P < 0.01, ***P < 0.001, ****P < 0.0001; significantly different (Student’s *t* test). Data are combined from 2 experiments.

Finally, given that hPBMC mouse–derived CD8^+^ T cells induce B cell apoptosis, the indirect regulation of Tph/Tfh cell differentiation and proliferation was examined via B cells. CD8T/BΔhPBMC mice were created by depleting CD8^+^ T and CD19^+^ B cells from PBMCs and examining the effects on Tph/Tfh cell skewing (Figure 9A). CD8^+^ T cells and CD19^+^ B cells were depleted by magnetic beads from PBMCs, and we confirmed the depletion by flow cytometry (Supplemental Figure 11). Importantly, B cell removal significantly reduced SjS-related factors induced in CD8T/BΔhPBMC mice, including IgG, ANA, and anti-SSA antibody (Figure 9B). A significant reduction was also shown in CD4^+^ T cell and Tph/Tfh cell numbers in the spleen of CD8T/BΔhPBMC mice compared with CD8TΔhPBMC mice (Figure 9C). However, the proportion of Tph/Tfh cells within CD4^+^ T cells was unaffected, revealing that while B cells influence the increase in CD4^+^ T cells in CD8TΔhPBMC mice, they do not affect the specific increase in Tph/Tfh cells (Figure 9D). This indicates that B cells regulate CD4^+^ T cells, including Tph/Tfh cells, in CD8T/BΔhPBMC mice. However, the direct action of CD8^+^ T cells is responsible for the polarization of Tph/Tfh cells in hPBMC mice. Furthermore, in this model, it has become evident that B cells indirectly exacerbate SjS pathogenesis by increasing Tph cells and producing SjS-related factors, such as anti-SSA antibodies.

## Discussion

Tph cells are currently a well-defined population only in humans, and similar cells have not been reported in rodents such as mice. The fact that CXCL13, a crucial chemokine for Tph cell function, is primarily sourced from stromal cells in mice suggests that Tph cells arise from human-specific mechanisms (22–27). Therefore, in this study, we developed a mouse model that allows in vivo proliferation of human Tph, Tfh, and B cells and induces Tph cell–dependent SjS-like symptoms. This mouse model demonstrates utility as a potentially novel SjS model, showing not only lymphocytic infiltration into the salivary and lacrimal glands but also significant reduction in salivary gland function, along with elevated markers of SjS, including serum IgG, ANA, and anti-SSA and anti-SSB antibodies. It also replicates fundamental extraglandular symptoms of SjS, such as lung manifestations, enabling evaluation of therapeutic interventions for these conditions, which enhances the model’s value. The mechanism of disease onset involves direct tissue damage by Tph cells, as demonstrated by the results of in vitro transfer tests and cytotoxicity assays, which indicate that Tph cells exhibit characteristics of CD4^+^ CTLs, a concept of recent interest (41). Additionally, the model highlights the contribution of B cells to SjS pathogenesis through the induction of IgG, ANA, and anti-SSA antibody, as well as proliferative effects on Tph cells, reflecting clinical conditions. Furthermore, the increased Tfh cells in this model and their role in inducing B cells suggest that Tfh cells indirectly control Tph cells by regulating B cell numbers, potentially exacerbating the disease process.

Tph and Tfh cells induced in CD8TΔhPBMC mice markedly reflect the cellular characteristics observed in human pathology, in terms of gene and protein expression, demonstrating the model’s utility. Bulk RNA-Seq analysis using the spleen tissues of CD8TΔhPBMC mice showed distinct expression of transcription factors characteristic of these cells, such as PRDM1 and MAF in Tph cells and BCL6 in Tfh cells. Furthermore, Tph cells exhibited distinctive expression of ZEB2, a marker recently identified for distinguishing age-associated helper T (ThA) cells (41). This overlap between ThA and Tph cells is consistent with our findings. However, SOX4, which is highly expressed in Tph cells from patients with systemic lupus erythematosus (SLE) (2, 18), was not observed in Tph cells from CD8TΔhPBMC mice. Furthermore, scRNA-Seq and multicolor flow cytometry using the salivary gland cells revealed that although CXCL13^+^IL-21^+^ Tph cells expressed IFN-γ and perforin, they did not notably express granzyme. Conversely, other Tph-like fractions expressing high granzyme levels were observed in the salivary glands. This indicates that Tph cells involved in helping B cells and those inducing cytotoxicity are distinct populations. Further studies focusing on the differentiation and mechanisms and the relationship of these cells are necessary.

SjS is a notable autoimmune disease that requires further examination, and several therapeutic agents are currently being developed. Although some treatments like rituximab and abatacept have exerted limited efficacy, clinical development for several drugs, such as anti-CD40L/CD40 antibodies, anti–BAFF-R antibodies, and JAK inhibitors, is underway (30, 31, 42–44). As the targets of these drugs extensively overlap with factors controlling Tph/Tfh cells, Tph/Tfh cells are nevertheless promising targets. CD8TΔhPBMC mice allow stable assessment of reduced saliva production, which was challenging in traditional models such as NOD mice, and shows Tph cell infiltration in systemic organs beyond the glands. Therefore, drug effects on systemic symptoms may be evaluated using cell infiltration as an indicator. Furthermore, CXCL13 is elevated in the blood of CD8TΔhPBMC mice, which can serve as a marker indicating its effect on the extraglandular manifestations of SjS. CXCL13 has been reported to correlate with the ESSDAI, an evaluation index of extraglandular symptoms, and is commonly used in clinical trials (30, 42). Replacing Tph, Tfh, and B cells with human cells in this model also allows evaluation of biologics and small molecules that only crossreact with human targets. Generally, the SjS pathology in the salivary glands is sequentially induced by CD4^+^ T and B and CD8^+^ T cells. Thus, this model effectively reflects the early pathology of SjS. Reproducing the process of gland symptom exacerbation induced by CD8^+^ T cells remains challenging; however, methods such as reintroducing CD8^+^ T cells after establishing CD8^+^ T cell–depleted PBMCs in mice could be considered. The high similarity between CD4^+^ T cells from CD8TΔhPBMC mice and those proliferating in patients detected pathologically indicates a high degree of pathological reflection. CD8TΔhPBMC mice naturally develop SjS-like symptoms, which, as shown in Figure 6, can be exacerbated or potentially result in the construction of other disease models mediated by these cells with additional exogenous stimuli. IFN-α signaling is a known characteristic of patients with SjS and SLE and is important for Tph cell differentiation and activation (28, 45–49). Consistent with these findings, our study demonstrated that in vivo treatment with IFN-α activated Tph cells, indicating that this phenomenon reflects mechanisms observed in humans. Future studies should evaluate the impact of nucleic acid–responsive Toll-like receptor signaling, which induces type I IFNs, on pathology and the effects of antigen addition on antibody production.

Two well-known methods for creating humanized mouse models are transplanting human HSCs or human PBMCs into immunodeficient mice. HSC-transplanted humanized mice have the advantage of establishing myeloid cells and other lymphocyte populations besides T and B cells, but they require 4–5 months to develop, need host immune cell removal via radiation, and have variable cell engraftment and evaluation metrics (50–52). Conversely, PBMC-based humanized mice can be made quickly and stably within a few weeks. The traditional hPBMC mouse model was limited by the engraftment of only T cells and the GVHD development with human cell engraftment (53, 54). However, by depleting CD8^+^ T cells from PBMCs, the GVHD limitation was managed, enabling B cell engraftment. Although this model cannot examine mechanisms mediated by CD8^+^ T cells, it offers a significant advantage in creating mice that coengraft B, Tph, and Tfh cells in a short time. Furthermore, unlike HSC-transplanted mice, which can only induce T and B cells without TCR and B cell receptor (BCR) rearrangements, CD8^+^ T–depleted humanized mice can engraft T and B cells, preserving the donor’s TCR and BCR. Using patient-derived PBMCs allows examination of patient-specific T cells, B cells, and autoantibodies produced by B cells, facilitating further elucidation of human pathology using this technique.

In this study, we successfully developed an in vivo tool capable of addressing various challenges in studying human- and patient-specific Tph, Tfh, and B cells. This model naturally develops SjS-like symptoms and replicates various human autoimmune disease pathologies in vivo, paving the way for developing treatments for difficult-to-treat diseases.

## Methods

### Sex as a biological variable.

Regarding the sex of the hPBMC donors transferred into mice, we confirmed that similar phenomena were observed with PBMCs derived from both male and female donors. Regarding the sex of the recipient mice, only female mice were used in all experiments in this study in order to obtain reproducible data. Since both sexes are commonly used for the generation of PBMC-based humanized mice, it is presumed that the findings are likely applicable to mice of both sexes.

### Mice.

Female NSG (NOD.Cg-Prkdcscid Il2rgtm1Wjl/SzJ) mice were obtained from the Jackson Laboratory Japan and used for human cell transplantation studies at 6–7 weeks of age. Female NOG (NOD/Shi-scid, IL-2RγKO Jic) mice were obtained from In-vivo Science and used for human cell transplantation studies at 6 weeks of age. These mice were maintained under specific pathogen–free conditions in the Animal Care Facility of Mitsubishi Tanabe Pharma Corporation. All experiments involving animals were approved by the animal study committees of Mitsubishi Tanabe Pharma Corporation and performed according to institutional guidelines and Home Office regulations.

### Transplantation of human cells.

Donated hPBMCs were purchased from STEMCELL Technologies Canada. CD8^+^ T cell–depleted PBMCs were collected with anti-human CD8 magnetic beads using MACS columns (Miltenyi Biotec) following the manufacturer’s protocols as previously reported (37). B cells were depleted using anti-human CD19 magnetic beads following the manufacturer’s instructions (Miltenyi Biotec). PBMCs, CD8^+^ T cell–depleted PBMCs, or CD8^+^ T and B cell–depleted PBMCs were resuspended in phosphate-buffered saline (PBS) (Nacalai Tesque) and intravenously injected (1 × 10^7^ cells) into the tail veins of NSG mice. The day of cell transplantation was designated as day 0. The mice were sacrificed on days 27–31 after cell transplantation. A scoring system was used to monitor GVHD symptoms, yielding a total clinical score out of 10 (Supplemental Table 1). For generation of HCS (NOG-hCD34) mice, NOG mice at 6 weeks of age were irradiated with a nonlethal dose of x-rays (2 Gy). Human umbilical cord blood–derived CD34^+^ HSCs from Lonza were resuspended in PBS and intravenously injected (5 × 10^4^ cells) into the tail veins of NOG mice. Mice were sacrificed 16 weeks after cell transplantation.

For the Tph cell transfer experiment, cells were isolated from the splenocytes of CD8TΔhPBMC mice using a 2-step magnetic bead separation technique. First, Mouse CD45 MicroBeads, Human CD8 MicroBeads, and Human CD19 MicroBeads (all Miltenyi Biotec) were used for negative selection via a magnetic column. In the second step, the flow-through cells underwent a second round of negative selection using Biotin Anti-human CXCR5 Antibody (BioLegend), Streptavidin MicroBeads (Miltenyi Biotec), a Mouse CD4^+^ T Cell Isolation Kit (Miltenyi Biotec), and a Human CD4^+^ T Cell Isolation Kit (Miltenyi Biotec). The isolated cells were used as Tph cells for adoptive transfer.

The Institutional Review Board of Mitsubishi Tanabe Pharma Corporation approved all human studies according to the Declaration of Helsinki guidelines revised in 2008.

### Measurement of serum ALT activity.

Following the manufacturer’s instructions, serum ALT was measured with a DRI-CHEM analyzer (Fuji Film).

### Measurement of plasma IgG, anti-SSA antibody, anti-SSB antibody, and ANA activity.

Following the manufacturer’s instructions, plasma IgG levels were measured using Human Anti-dsDNA IgG High Sensitivity ELISA kit (M-3100HS, Alpha Diagnostic). For the detection of anti-SSA antibodies, the Anti-Sjögren syndrome type A antigen (SSA/Ro) IgG ELISA Kit (3210-SSA, Alpha Diagnostic) was used. Similarly, anti-SSB antibodies were measured using the Anti-Sjögren syndrome type B antigen (SSB/La) IgG ELISA Kit (3220-SSB, Alpha Diagnostic). For the detection of ANA, the ANA Screen ELISA Kit (KA 0939, Abnova) was used. Absorbance was read using an EnVision plate reader (PerkinElmer) and a SpectraMax ABS Plus microplate reader (Molecular Devices).

### Measurement of plasma SP-D.

Following the manufacturer’s instructions, plasma surfactant protein D was measured using Rat/Mouse SP-D kit YAMASA EIA (96 Reactions) Kit (Cosmo Bio). Absorbance was read using SpectraMax ABS Plus microplate reader (Molecular Devices).

### Measurement of plasma and urine glucose.

Following the manufacturer’s instructions, plasma and urine glucose was measured using glucose colorimetric assay kit (Cayman Chemical Company). Absorbance was read using SpectraMax ABS Plus microplate reader (Molecular Devices).

### Measurement of proteinuria.

Urinary protein concentration was measured using the Pierce Coomassie Plus (Bradford) Assay Kit (Thermo Fisher Scientific), following the manufacturer’s instructions. Urine samples were collected from individual mice by manual bladder expression. The collected urine samples were used for protein quantification. Briefly, 150 μL of Coomassie reagent was added to 5 μL of urine sample or bovine serum albumin (BSA) standard in a 96-well plate. After incubation at room temperature for 10 minutes, absorbance was measured at 595 nm using SpectraMax ABS Plus microplate reader (Molecular Devices). Protein concentrations were calculated from the BSA standard curve and expressed (mg/mL).

### Isolation of mouse tissue-derived immune cells.

The spleen and salivary gland tissues were collected from hPBMC, hPBMCΔCD8T, or hPBMCΔCD8T/B mice. Spleens were mechanically dissociated using gentle MACS dissociator (Miltenyi Biotec) and then lysed using HLB solution (Immuno-Biological Laboratories) to remove red blood cells and obtain cell suspensions of mononuclear cells. Half the salivary gland tissues were transferred to GentleMACS C tube (Miltenyi Biotec) containing 2 mL digestion buffer (RPMI 1640, Merck) supplemented with 10% FBS (Gibco), 0.5–1 mg/mL Collagenase (Fuji Film Wako Pure Chemical Corporation), and 0.1 mg/mL DNase I (Roche Diagnostics). Tissues were mechanically dissociated into small fragments using the GentleMACS Octo dissociator (Miltenyi Biotec). Samples were then incubated at 37°C for 45 minutes with continuous rotation to enhance enzymatic digestion. After incubation, tissue suspensions were allowed to further dissociate using the GentleMACS Octo dissociator. The resulting suspensions were diluted with RPMI, transferred to 15 mL conical tubes, and centrifuged at 340*g* for 5 minutes. After discarding the supernatant, cell pellets were resuspended in 5 mL 40% Percoll (Cytiva Sweden AB). A density gradient was created with 2 mL 75% Percoll gently layered beneath the 40% Percoll solution. The gradient was centrifuged at 700–800*g* for 20 minutes at 20°C. The mononuclear cells in the middle layer were carefully collected. Finally, the cells were resuspended in FACS buffer (Thermo Fisher Scientific) and used for flow cytometry and cell sorting.

Cell isolation from the skin, pancreas, and kidney was performed using the same method as that for the salivary gland tissues. sLN and lung tissues were mechanically dissociated with sandwiching the organ between glass slides and gently rubbing them together to prepare the single cells. Small and large intestinal lamina propria mononuclear cells were collected as previously (55). Briefly, dissected small intestine or large intestine mucosa was cut into 5 mm pieces. Tissue was incubated with HBSS containing 1 mM DTT and 5 mM EDTA at 37°C for 30 minutes, the remaining supernatant was centrifuged at 340*g* for 5 minutes, and the pellet was obtained. The pellet was digested in RPMI 1640 medium (Sigma-Aldrich) containing 1 mg/mL collagenase (Fuji Film Wako Pure Chemical Corporation) and 0.1 mg/mL DNase (Roche Diagnostics) and 10% FBS for 30 minutes. Density gradient centrifugation was performed using the dissolved solution as described above. Bone marrow cells were isolated from the tibias. Bones were dissected and cleaned of muscle tissue, then rinsed in PBS. The bone marrow was flushed out using a 25–27G needle attached to a syringe filled with RPMI 1640. The resulting cell suspension was filtered through a 70 μm cell strainer (Miltenyi Biotec), then lysed using HLB solution to remove red blood cells and obtain cell suspensions of mononuclear cells.

### Flow cytometry.

After blocking with an anti-human FcR antibody (TruStain FcX, BioLegend) for 5 minutes, mouse tissue-derived cells or in vitro–cultured cells were incubated with specific fluorescence-labeled antibodies at 4°C for 20 minutes. The following antibodies and dyes were used for conventional flow cytometry: anti-human CD45 (PE/Cyanine7, clone 2D1; BioLegend), anti-human CD3 (BV605, clone UCHT1; BioLegend), anti-human CD8a (BV510, clone RPA-T8; BioLegend), anti-human CD4 (PerCP/Cyanine5.5, clone OKT4; BioLegend), anti-human CD185 (CXCR5) (APC, clone J252D4; BioLegend), anti-human CD279 (PD-1) (BV421, clone MIH4, BD Biosciences), anti-human CD19 (BV421, clone HIB19; BD Biosciences), and Fixable Viability Dye eFluor 780 (Thermo Fisher Scientific).

For spectral flow cytometry, mouse tissue-derived cells were incubated with specific fluorescence-labeled antibodies at 37°C for 15 minutes. The following antibodies and dyes were used: anti-human CD45 (BUV496, clone HI30; BD Biosciences), anti-human CD3 (PE/Cyanine5, clone HIT3a; BioLegend), anti-human CD3 (PE/Cyanine7, clone UCHT1; BioLegend), anti-human CD8 (BUV805, clone SK1; BD Biosciences), anti-human CD4 (Spark Blue 550, clone SK3; BioLegend), anti-human HLA-DR (Alexa Fluor 700, clone LN3; BioLegend), anti-human CD200 (BUV615, clone OX-104; BD Biosciences), anti-human CD127 (BV650, clone A019D5; BioLegend), anti-human/mouse/rat CD278 (ICOS) (FITS, clone C398.4A; BioLegend), anti-human CD38 (BV510, clone HB-7; BioLegend), anti-human CD279 (PD-1) (PE/Fire 700, clone A17188B; BioLegend), anti-human TIGIT (VSTM) (PE/Fire, clone A15153G; BioLegend), anti-human CD185 (CXCR5) (Biotin, clone J252D4; BioLegend), streptavidin (BV421, RUO; BioLegend), anti-human CD19 (PE/Cyanine5, clone HIB19; BioLegend), anti-human CD38 (FITC, clone HB-7; BioLegend), anti-human CD138 (Syndecan-1) (BV605, clone MI15; BioLegend), anti-human CD192 (CCR2) (PE/Dazzle 594, clone KO36C2; BioLegend), anti-human CD154 (BV605, clone 24-31; BioLegend), anti-mouse CD45 (BUV563, clone 30-F11; BD Biosciences), anti-mouse FcR antibody (TruStain FcX, BioLegend), anti-human FcR antibody (TruStain FcX, BioLegend), and Zombie Dye NIR (BioLegend). For the detection of plasmablasts, plasma cells, and non–T cells/B cells, the following antibodies and dyes were used: anti-human CD19 (BV421, HIB19; BioLegend), anti-human IgD (PE/Dazzle 594, IA6-2; BioLegend), anti-human CD27 (BV750, O323; BioLegend), anti-human CD38 (FITC, HIT2; BioLegend), anti-human CD138 (BV605, MI15; BioLegend), and anti-human CD3 (PE/Cyanine5, clone HIT3a; BioLegend). To perform additional intracellular staining, the cells were fixed with CytoFix (BD Biosciences) at room temperature for 20 minutes, followed by washing with 1× CytoPerm (BD Biosciences). Anti-human granzyme A (PerCP/Cyanine5.5, clone CB9; BioLegend), anti-human/mouse granzyme B (APC/Fire 750, clone QA16A02; BioLegend), and CXCL13 monoclonal (PE, clone 53610; Thermo Fisher Scientific) antibodies were added to the permeabilized cells and incubated at room temperature for 30 minutes. Then, the cells were resuspended in 80–150 μL of FACS buffer or permeabilization buffer and analyzed for flow cytometry.

In the case of additional intracellular cytokine staining with stimulation, the cells were stimulated using the Cell Stimulation Cocktail (Thermo Fisher Scientific) and GolgiStop Protein Transport Inhibitor (BD Biosciences) diluted in complete RPMI (consisting of RPMI 1640 supplemented with 10% FBS, 1% penicillin-streptomycin, 10 mM HEPES, 1× MEM–nonessential amino acids, and 55 μM 2-mercaptoethanol) for 3–4 hours at 37°C. Following the stimulation, cell surface and intracellular staining were performed as described above. Anti-human IL-21 (APC, clone 3A3-N2; BioLegend) and anti-human IFN-γ (BUV395, clone B27; BD Biosciences) were added to the permeabilized cells and incubated at room temperature for 30 minutes. Then, the cells were resuspended in 80–150 μL of FACS buffer or permeabilization buffer and analyzed for flow cytometry.

Conventional flow cytometry was performed using a Fortessa X-20 (BD Biosciences) and analyzed using FlowJo software (Tree Star), whereas spectral flow cytometry was performed using an ID7000 Spectral Cell Analyzer (Sony) and analyzed as described below. Cell sorting was performed using FACSAria III (BD Biosciences) with confirmation of more than 95% purity of the sorted cells.

For multiparameter flow cytometry analysis, FlowJo software was first used to curate FCS files containing flow cytometry data, and human CD4^+^ T cells were defined as presented in Supplemental Figure 2. After manual scaling for each marker on FlowJo, expression values for each cell were exported as a count matrix. The count matrix and metadata were integrated using the tidytof package (1.0.0) in the R package. The number of cells for analysis was downsampled to a maximum of 10,000 per tissue. A total of 110,531 cells from 14 tissues of the whole PBMC- and CD8ΔPBMC-transferred mice were subjected to clustering. After clustering using the FlowSOM package (2.14.0) and UMAP dimensional reduction using the uwot package (0.2.2), cell clusters were visualized with tidytof. Each cluster was annotated based on marker expression patterns. GraphPad Prism (GraphPad Software) was used to perform data visualization for cluster frequency.

### Bulk RNA-Seq.

RNA-Seq was performed on isolated cells described in *Flow cytometry* section. Total RNA was isolated from each sample using RNeasy Micro Kit (QIAGEN). The integrity and quantity of the total RNA were measured with a 2100 Bioanalyzer RNA 6000 Pico Kit (Agilent Technologies). Library preparation was performed using a NEBNext Ultra II Directional RNA Library Prep Kit for Illumina (New England Biolabs) with NEBNext rRNA Depletion Kit v2 (Human/Mouse/Rat) (New England Biolabs) following the manufacturer’s instructions. The quality of the libraries was assessed using a 2200 TapeStation High Sensitivity D1000 (Agilent Technologies). The equally pooled libraries of the samples were sequenced using the Illumina NextSeq 500 in 76 bp single-end reads. Sequencing adaptors, low-quality reads, and bases were trimmed with Trimmomatic-0.39 tool (56). The sequence reads were aligned to the human reference genome (hg38) using STAR 2.7.9a (57). The aligned reads were subjected to downstream analyses using StrandNGS 4.0 software (Agilent Technologies). The read counts allocated for each gene and transcript (Ensembl Database 2016.12.01) were quantified using a TPM method (58, 59). Heatmap was generated using the R package.

### ScRNA-Seq.

Human CD45^+^ cells were sorted from the pooled cells of 4 mice, and approximately 13,500 cells with more than 95% survival rate were loaded into a Chromium Chip K to generate single-cell emulsion using Chromium Next GEM Single Cell 5′ Kit v2 and a Chromium Controller (10x Genomics). RNA-Seq libraries were then prepared using Chromium Next GEM Single Cell 5′ Kit v2 following the manufacturer’s instructions (10x Genomics). The generated scRNA-Seq libraries were sequenced using a NovaSeq 6000 platform (Illumina).

Sequence reads from all samples were processed using Cell Ranger (10x Genomics). Seurat version 5.1.0 was used to aggregate and analyze the processed data by following the official vignettes (https://satijalab.org/seurat/articles/seurat5_integration). Quality control was first performed, filtering out cells with more than 20% mitochondrial reads and with < 200 or > 6,000 reads. Cells that passed the quality control were normalized. After normalization, specific filtering criteria were applied to subset CD4^+^ T cells. Specifically, cells were selected based on the following criteria: CD3D expression, > 0.2; CD14, < 0.3; CD8A, < 0.25; CD8B, < 0.2; FCGR3A, < 0.2; CD1C, < 0.3; and S100A8, < 0.5. Cells that passed filtering were scaled, and principal component analysis was performed. To account for sample variation, the Seurat object for each sample was individually integrated using Anchor-based RPCA integration, where each Seurat object was corrected by the sample. Eleven clusters (0–10) were projected on UMAP space with the integrated data: (dims = 1:50), FindNeighbors (dims = 1:50), and FindClusters (resolution = 0.5). A gene expression heatmap showing the unbiased generation of the top 10 differentially expressed genes for each cluster was generated using the FindAllMarkers function. Trajectory and pseudotime analysis were performed with Monocle3 using a Seurat object from which clusters 9 (non-CD4^+^ T cells) and 11 (regulatory T cells) were removed. The root node of the trajectory was defined based on cluster 3 (naive-like cluster).

### Drug administration.

Tacrolimus (Prograf capsules 5 mg) (Astellas) was orally administered once a day from day 15 at a dose of 10 mg/kg. Cytokine mixture (recombinant human IL-12 p70 from PeproTech), recombinant human interferon-α (pbl Assay Science), and recombinant human TGF-β1 (PeproTech) were subcutaneously administered 3 times a week from day 4 at a dose of 1 μg (each)/100 μL/head.

### Evaluation of salivary flow rate.

Mice were anesthetized by subcutaneously injecting a mixture of medetomidine (Kyoritsu Seiyaku), midazolam (Sandoz K.K.), and butorphanol (Meiji Seika) (0.15, 2, 2.5 mg/kg, respectively). Mice under anesthesia were stimulated with pilocarpine (Fuji Film Wako Pure Chemical Corporation) intraperitoneally (0.5 mg/kg). A micropipette was used to gravimetrically collect stimulated whole saliva for 20 minutes at room temperature. After collecting saliva, the mice were intraperitoneally administered with atipamezole (α2 adrenergic receptor antagonist, Kyoritsu Seiyaku, 0.15 mg/kg) and upon regaining consciousness were returned to their cages.

### Histological analysis.

For histological analysis, submandibular glands were fixed with 10% buffered formalin (Fuji Film Wako Pure Chemical Corporation) and embedded in paraffin. Tissue sections were stained with hematoxylin and eosin. Each staining step was performed at room temperature. Samples were evaluated under the BX-53 fluorescence microscope (Evident).

For CD3 immunohistochemical staining, slides were subjected to heat-induced epitope retrieval in Tris-EDTA solution. Endogenous peroxidase in tissues was blocked by incubation of slides in 3% hydrogen peroxide solution before incubation with primary antibody anti-CD3 (AbD Serotec catalog MCA1477). After being incubated overnight at 4°C, primary Abs were followed by ImmPRESS Reagent Anti-Rat Ig (Vector Laboratories catalog MP-7444) for 30 minutes. Peroxidase Stain DAB Kit (Nacalai) was used to develop DAB chromogen, and hematoxylin staining solution was used to stain nuclei. According to the severity of various lesions, the qualitative score was given as follows: –, not remarkable; +/–, minimal; +, slight; 2+, moderate; 3+, severe.

### Measurement of plasma CXCL13 concentration.

CXCL13 concentration in the samples was determined using the AlphaLISA Human CXCL13 Detection kit (Revvity) following the manufacturer’s instructions. Luminescence was detected using the EnVision plate reader (PerkinElmer, Revvity). Cytokine concentration was quantified using a standard curve. Data were analyzed using GraphPad Prism (GraphPad Software).

### In vitro CD8^+^ T or B or CD4^+^ T cell coculture assay.

CD8^+^ T cells were isolated from hPBMC-transferred NSG mice. B, CD4^+^ T, and control CD8^+^ T cells were isolated from hPBMCs. In brief, PBMCs were intravenously injected into NSG mice as described above. On day 21 after injection, splenocytes were isolated from the mice. Splenocytes were stained with Zombie NIR Fixable Viability Kit (BioLegend), anti-mouse FcR antibody (TruStain FcX, BioLegend), and anti-human FcR antibody (TruStain FcX, BioLegend) at 4°C for 15 minutes to block nonspecific binding. For CD8^+^ T cell detection, cells were incubated with FITC anti-human CD8 antibody (BioLegend) and PE/Cyanine7 anti-human CD3 antibody (BioLegend) at 4°C for 20 minutes. CD8^+^ T cells derived from hPBMC-transferred NSG mice and control CD8^+^ T cells were defined as CD3^+^CD8^+^ cells and sorted using a FACSAria III cell sorter (BD Biosciences). For B and CD4^+^ T cells, PBMCs were obtained from healthy donors. B cells were isolated using the B-Cell Isolation Kit II, human (Miltenyi Biotec), and CD4^+^ T cells were isolated using the CD4^+^ T Cell Isolation Kit, human (Miltenyi Biotec), following the manufacturer’s protocols.

CD8^+^ T cells derived from hPBMC-transferred NSG mice or control CD8^+^ T cells (5 × 10^4^) were cultured with either B (1 × 10^4^) or CD4^+^ T cells (1 × 10^4^) in 96-well, round-bottom plates precoated with anti-CD3 antibody (5 μg/mL; clone OKT3, BioLegend). Cells were cultured in complete RPMI containing anti-CD28 antibody (2 μg/mL; clone CD28.2, BioLegend). For coculture with B cells, BAFF (200 ng/mL; PeproTech) was added to the medium. In the inhibition assay, neutralizing antibodies or inhibitors were added to the medium at 2 concentrations. Evaluated neutralizing antibodies or inhibitors were Anti-Fas (ZB4; MBL) InVivoMAb anti-human IFNγ (B27; Bio X Cell), InVivoSIM anti-human TNFα (Infliximab; Bio X Cell), Purified anti-human CD253 (TRAIL) Antibody (RIK-2; BioLegend), and Granzyme B Inhibitor Z-AAD-CH2Cl (GLPBIO). Purified Mouse IgG1 and Purified Human IgG1 (BioLegend) were used as isotype controls. Dimethyl sulfoxide (Kanto Kagaku) was used as the vehicle control for Granzyme B Inhibitor. Cultures were maintained at 37°C in a humidified incubator with 5% CO_2_ for 2 days. The Zombie NIR Fixable Viability Kit (BioLegend) in combination with anti-human FcR antibody (TruStain FcX, BioLegend) was used to stain late apoptotic cells at 4°C for 15 minutes following the manufacturer’s instructions. For cell surface marker staining, cells were incubated with fluorescence-labeled antibodies at 4°C for 20 minutes. After washing, early and late apoptotic cells were identified by staining with PE/Cyanine7 Annexin V (BioLegend). Data were acquired using the ID7000 Spectral Cell Analyzer.

### Evaluation of Tph cell cytotoxicity.

The same Tph cells used for the Tph cell transfer experiment were used in the cytotoxicity assay. Briefly, mouse CD45^+^ cells were isolated from the spleen and bone marrow of CD8TΔhPBMC mice for use as target cells. Tph cells (1 × 10^5^) were cultured with either mouse CD45^+^ splenocytes (1 × 10^4^) or mouse CD45^+^ bone marrow cells (1 × 10^4^) in 96-well, round-bottom plates precoated with anti-CD3 antibody (10 μg/mL; clone OKT3, BioLegend). The cells were cultured in complete RPMI medium containing anti-CD28 antibody (5 μg/mL; clone CD28.2, BioLegend) and murine GM-CSF (10 ng/mL; PeproTech) for 16 hours at 37°C in a humidified incubator under 5% CO_2_. Flow cytometry was performed using the same method as described for the in vitro coculture assay of CD8^+^ T and B and CD4^+^ T cells. The percentage of dead cells was defined as the proportion of target cells that stained positive for either Zombie or annexin V.

### Statistics.

Statistical analyses were performed using GraphPad Prism (version 10, GraphPad Software). Differences between 2 groups were evaluated using the 2-sided unpaired Student’s *t* test. Comparisons of more than 2 groups were performed using 1-way ANOVA, followed by Tukey-Kramer multiple-comparison post hoc test. For all analyses, significance was accepted at the 95% confidence interval level (*P* < 0.05). The analysis was conducted using parametric analysis assuming a normal distribution.

### Study approval.

All experiments involving animals were approved by the regional animal study committees (Mitsubishi Tanabe Pharma Corporation) and were performed according to institutional guidelines and Home Office regulations. The institutional review board of Mitsubishi Tanabe Pharma Corporation approved all human studies according to the guidelines of the 1975 Declaration of Helsinki (2008 revision). We purchased and used samples that were obtained from consented donors from a supplier.

### Data availability.

The raw bulk RNA-Seq and scRNA-Seq data have been deposited in the NCBI GEO database under accession numbers GSE308690 and GSE308691. The data values associated with the main manuscript and supplement are available in the Supporting Data Values Excel file.

## Author contributions

MP, SF, and RS performed most experiments, analyzed the data, had scientific discussions, and supported preparation of the manuscript. YI performed scRNA-Seq data analysis. KT, SM, and AN performed several experiments. YK conceived and designed the study, performed experiments, analyzed the data, prepared the manuscript, and supervised the study.

## Funding support

- Mitsubishi Tanabe Pharma Corporation.

## Supplementary Material

Supplemental data

Supporting data values

## Version 1 11/24/2025

Electronic publication

## References

[1] Huang Y (2023). T peripheral helper cells in autoimmune diseases: What do we know?. Front Immunol.

[2] Yoshitomi H (2024). Peripheral helper T cells, mavericks of peripheral immune responses. Int Immunol.

[3] Haynes NM (2007). Role of CXCR5 and CCR7 in follicular Th cell positioning and appearance of a programmed cell death gene-1high germinal center-associated subpopulation. J Immunol.

[4] Moser B (2015). CXCR5, the defining marker for follicular B helper T (TFH) cells. Front Immunol.

[5] Rao DA (2017). Pathologically expanded peripheral T helper cell subset drives B cells in rheumatoid arthritis. Nature.

[6] Yabe H (2021). Cytotoxic Tph-like cells are involved in persistent tissue damage in IgG4-related disease. Mod Rheumatol.

[7] Yoshitomi H, Ueno H (2021). Shared and distinct roles of T peripheral helper and T follicular helper cells in human diseases. Cell Mol Immunol.

[8] Bocharnikov AV (2019). PD-1hiCXCR5- T peripheral helper cells promote B cell responses in lupus via MAF and IL-21. JCI Insight.

[9] Akiyama M (2023). T follicular helper cells and T peripheral helper cells in rheumatic and musculoskeletal diseases. Ann Rheum Dis.

[10] Zou X (2024). Peripheral helper T cells in human diseases. J Autoimmun.

[11] Del Carmen Crespo Oliva C (2024). T peripheral helper (Tph) cells, a marker of immune activation in cancer and autoimmune disorders. Clin Immunol.

[12] Harada T (2023). Peripheral helper-T-cell-derived CXCL13 is a crucial pathogenic factor in idiopathic multicentric Castleman disease. Nat Commun.

[13] Seyedsadr M (2024). A pathologically expanded, clonal lineage of IL-21-producing CD4^+^ T cells drives inflammatory neuropathy. J Clin Invest.

[14] Steere AC, Lemieux JE (2024). Wider recognition and greater understanding of postinfectious, antibiotic-refractory Lyme arthritis. J Clin Invest.

[15] Yong L (2021). Expanded circulating peripheral helper T cells in primary biliary cholangitis: Tph cells in PBC. Mol Immunol.

[16] Seki N (2024). Cytotoxic Tph subset with low B-cell helper functions and its involvement in systemic lupus erythematosus. Commun Biol.

[17] Schmitt N (2014). The cytokine TGF-β co-opts signaling via STAT3-STAT4 to promote the differentiation of human TFH cells. Nat Immunol.

[18] Shan Y (2023). TGF-β3 in differentiation and function of Tph-like cells and its relevance to disease activity in patients with systemic lupus erythematosus. Rheumatology (Oxford).

[19] McCarron MJ, Marie JC (2014). TGF-β prevents T follicular helper cell accumulation and B cell autoreactivity. J Clin Invest.

[20] Wallin EF (2014). Human T-follicular helper and T-follicular regulatory cell maintenance is independent of germinal centers. Blood.

[21] Baumjohann D (2013). Persistent antigen and germinal center B cells sustain T follicular helper cell responses and phenotype. Immunity.

[22] Ukita M (2022). CXCL13-producing CD4^+^ T cells accumulate in the early phase of tertiary lymphoid structures in ovarian cancer. JCI Insight.

[23] Dubey LK (2019). IL-4Rα-expressing B cells are required for CXCL13 production by fibroblastic reticular cells. Cell Rep.

[24] Kim CH (2004). Unique gene expression program of human germinal center T helper cells. Blood.

[25] Rasheed AU (2006). Follicular B helper T cell activity is confined to CXCR5(hi)ICOS(hi) CD4 T cells and is independent of CD57 expression. Eur J Immunol.

[26] Fujita N (2004). MTA3 and the Mi-2/NuRD complex regulate cell fate during B lymphocyte differentiation. Cell.

[27] Gunn MD (1998). A B-cell-homing chemokine made in lymphoid follicles activates Burkitt’s lymphoma receptor-1. Nature.

[28] Brito-Zeron P (2016). Sjögren syndrome. Nat Rev Dis Primers.

[29] Ramos-Casals M (2020). EULAR recommendations for the management of Sjögren’s syndrome with topical and systemic therapies. Ann Rheum Dis.

[30] Baer AN (2021). Efficacy and safety of abatacept in active primary Sjögren’s syndrome: results of a phase III, randomised, placebo-controlled trial. Ann Rheum Dis.

[31] Seror R (2021). Current and future therapies for primary Sjogren syndrome. Nat Rev Rheumatol.

[32] Chen W (2022). Tph cells expanded in primary Sjögren’s syndrome. Front Med (Lausanne).

[33] Hinrichs AC (2023). CCR9/CXCR5 co-expressing CD4 T cells are increased in primary Sjögren’s syndrome and are enriched in PD-1/ICOS-expressing effector T cells. Int J Mol Sci.

[34] Pontarini E (2020). Unique expansion of IL-21^+^ Tfh and Tph cells under control of ICOS identifies Sjögren’s syndrome with ectopic germinal centres and MALT lymphoma. Ann Rheum Dis.

[35] Scuron MD (2019). Spontaneous model of Sjögren’s Syndrome in NOD mice. Curr Protoc Pharmacol.

[36] Serreze DV (2024). Advancing animal models of human type 1 diabetes. Cold Spring Harb Perspect Med.

[37] Nishidate A (2025). Human PBMC-based humanized mice exhibit myositis features and serve as a drug evaluation model. Inflamm Regen.

[38] Sondergaard H (2013). Human T cells depend on functional calcineurin, tumour necrosis factor-α and CD80/CD86 for expansion and activation in mice. Clin Exp Immunol.

[39] Ito R (2013). Establishment of a human allergy model using human IL-3/GM-CSF-transgenic NOG mice. J Immunol.

[40] Argyriou A (2022). Single cell sequencing identifies clonally expanded synovial CD4^+^ T_PH_ cells expressing GPR56 in rheumatoid arthritis. Nat Commun.

[41] Goto M (2024). Age-associated CD4^+^ T cells with B cell-promoting functions are regulated by ZEB2 in autoimmunity. Sci Immunol.

[42] Fisher BA (2024). Safety and efficacy of subcutaneous iscalimab (CFZ533) in two distinct populations of patients with Sjögren’s disease (TWINSS): week 24 results of a randomised, double-blind, placebo-controlled, phase 2b dose-ranging study. Lancet.

[43] Gupta S (2024). Inhibition of JAK-STAT pathway corrects salivary gland inflammation and interferon driven immune activation in Sjögren’s disease. Ann Rheum Dis.

[44] Gandolfo S, Ciccia F (2022). JAK/STAT pathway targeting in primary Sjögren syndrome. Rheumatol Immunol Res.

[45] Del Papa N (2021). The role of interferons in the pathogenesis of Sjögren’s syndrome and future therapeutic perspectives. Biomolecules.

[46] Anders HJ (2020). Lupus nephritis. Nat Rev Dis Primers.

[47] Tanemura S (2022). Role of interferons (IFNs) in the differentiation of T peripheral helper (Tph) cells. Int Immunol.

[48] Tanemura S (2022). Role of interferons (IFNs) in the differentiation of T peripheral helper (Tph) cells. Int Immunol.

[49] Law C (2024). Interferon subverts an AHR-JUN axis to promote CXCL13^+^ T cells in lupus. Nature.

[50] Shultz LD (2007). Humanized mice in translational biomedical research. Nat Rev Immunol.

[51] Ishikawa F (2005). Development of functional human blood and immune systems in NOD/SCID/IL2 receptor γ chain^null^ mice. Blood.

[52] Hiramatsu H (2003). Complete reconstitution of human lymphocytes from cord blood CD34^+^ cells using the NOD/SCID/gammacnull mice model. Blood.

[53] Ito M (2002). NOD/SCID/gamma(c) (null) mouse: an excellent recipient mouse model for engraftment of human cells. Blood.

[54] Ehx G (2024). Pathophysiology and preclinical relevance of experimental graft-versus-host disease in humanized mice. Biomark Res.

[55] Miyamoto K (2023). The gut microbiota-induced kynurenic acid recruits GPR35-positive macrophages to promote experimental encephalitis. Cell Rep.

[56] Bolger AM (2014). Trimmomatic: a flexible trimmer for Illumina sequence data. Bioinformatics.

[57] Dobin A (2013). STAR: ultrafast universal RNA-seq aligner. Bioinformatics.

[58] Li B, Dewey CN (2011). RSEM: accurate transcript quantification from RNA-Seq data with or without a reference genome. BMC Bioinformatics.

[59] Wagner GP (2012). Measurement of mRNA abundance using RNA-seq data: RPKM measure is inconsistent among samples. Theory Biosci.

