# Supplementary material for: CD8+ T cell depletion promotes human Tph/Tfh cell proliferation and Sjögren syndrome–like symptoms in PBMC-based humanized mice
Source: JCI Insight. 2025 Nov 24;10(22):e191700. doi: 10.1172/jci.insight.191700 (PMC12643515; doi:10.1172/jci.insight.191700)
Supplement: Supplemental data [file jciinsight-10-191700-s060.pdf]

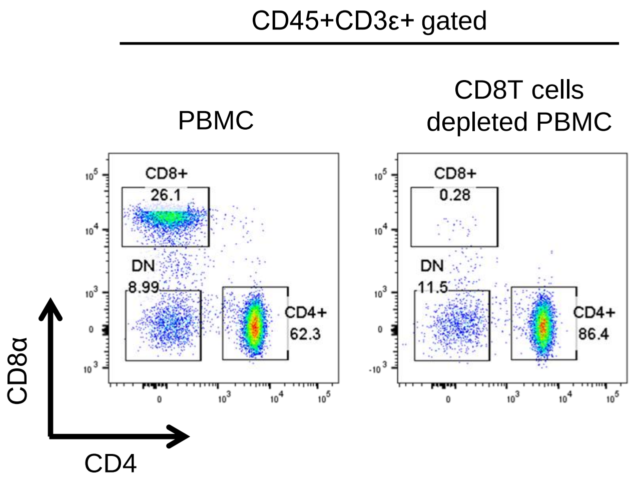

Supplementary Figure 1. Representative FACS plot of pre-transfer PBMC for hPBMC and CD8TΔhPBMC mice.

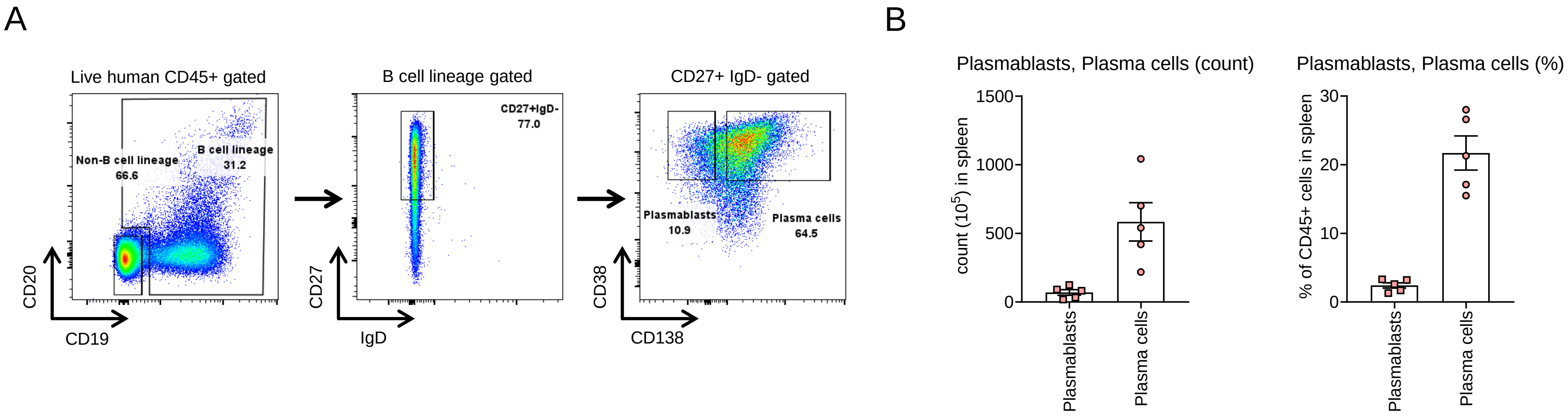

Supplementary Figure 2. The number and percentage of plasmablasts and plasma cells in the spleen of CD8TΔhPBMC mice.

(A) Representative FACS plot of CD20/ CD19, CD27/IgD, and CD38/CD138. (B) The number (left) and the percentage (right) of plasmablasts and plasma cell in the spleen of CD8TΔhPBMC mice. Data are presented as means with SEM (n = 5).

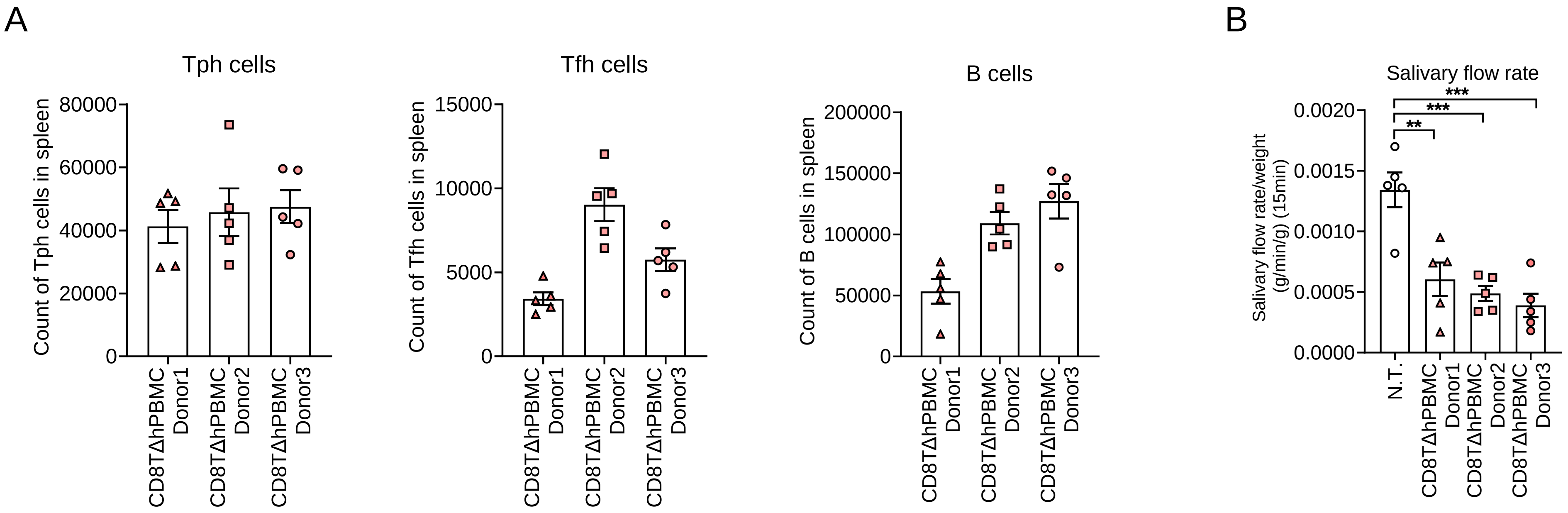

Supplementary Figure 3. Profile of immunological phenotype and salivary gland symptoms in CD8TΔhPBMC mice using different donors.

NSG mice were intravenously injected with human CD8T cell-depleted PBMCs from three individual donors. Mice were sacrificed on day 26. (A) The number of Tph cells, Tfh cells and B cells in the spleen. (B) Salivary flow rate was measured on day 20. Data are presented as means with SEM (n = 5). $^{**}P < 0.01$, $^{***}P < 0.001$. Statistical analysis was performed using one way ANOVA with Tukey's multiple-comparison post hoc test .

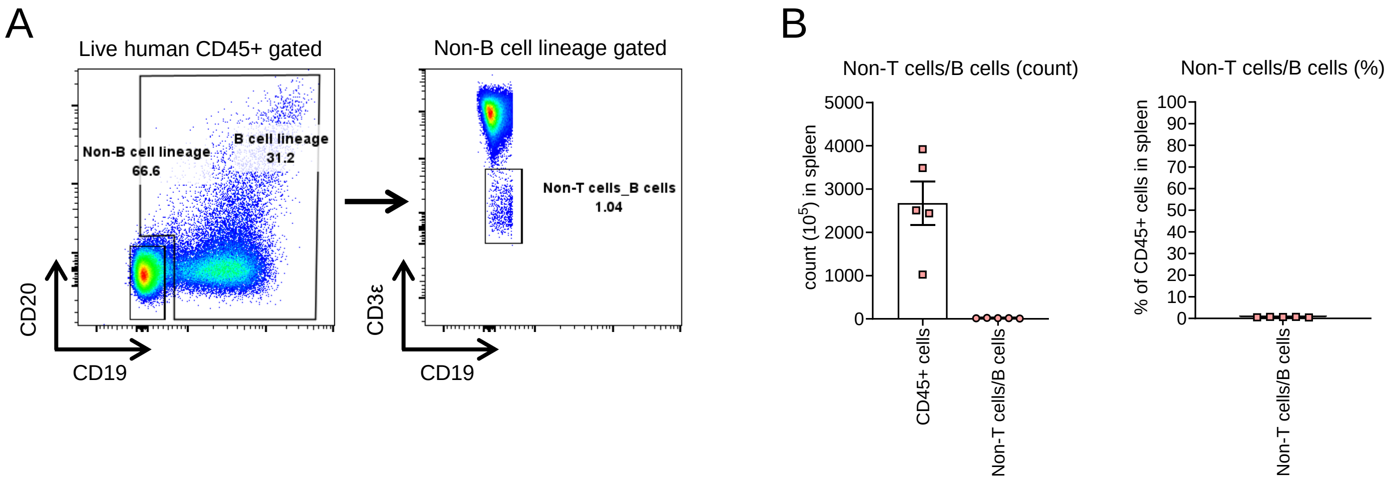

Supplementary Figure 4. The number and percentage of Non-T cells/B cells in spleen of CD8TΔhPBMC mice. (A) Representative FACS plot of CD20/ CD19 and CD3ε/CD19 staining of non-T cells/B cells. (B) The number (left) and percentage of non-T cells/B cells (right) in the spleen of CD8TΔhPBMC mice. Data are presented as means with SEM (n = 5).

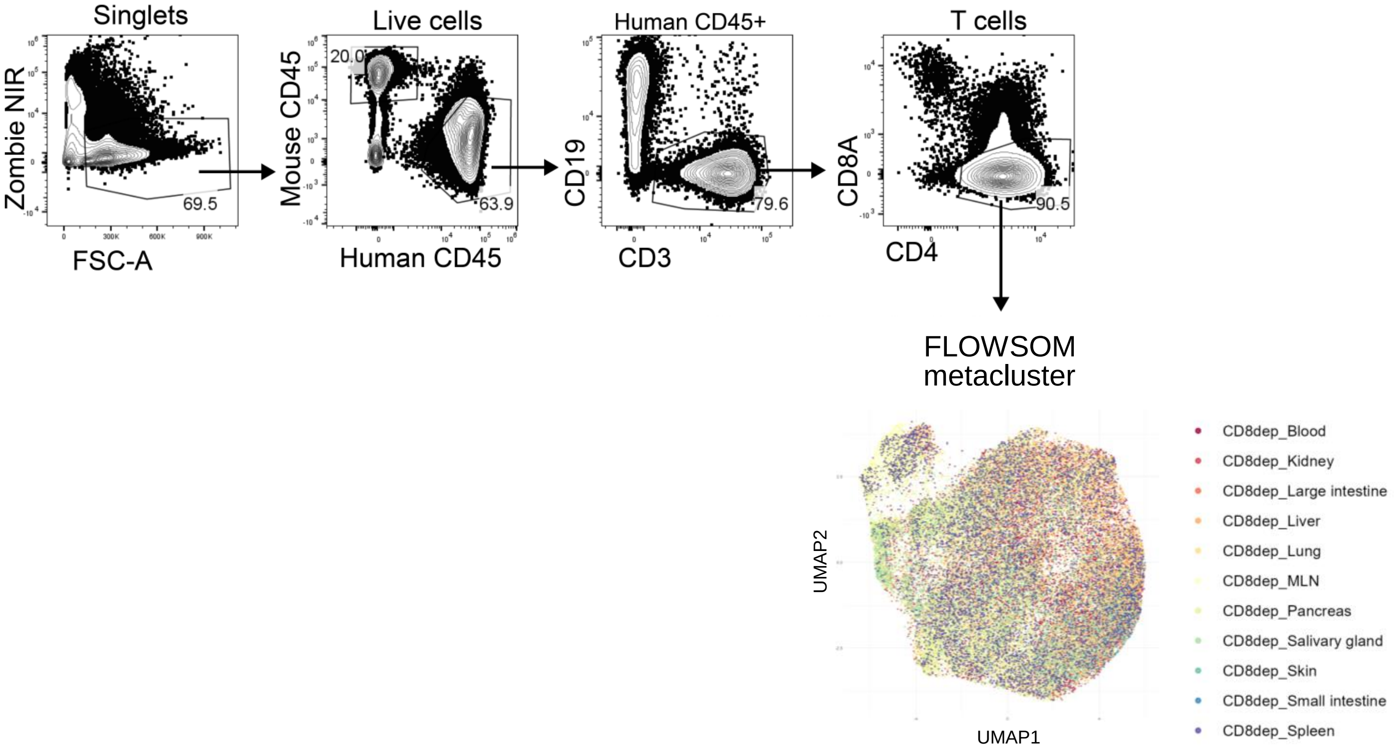

Supplementary Figure 5. Gating strategy of multi-parameter flow cytometry for CD4T cells in various organs of CD8TΔhPBMC mice.

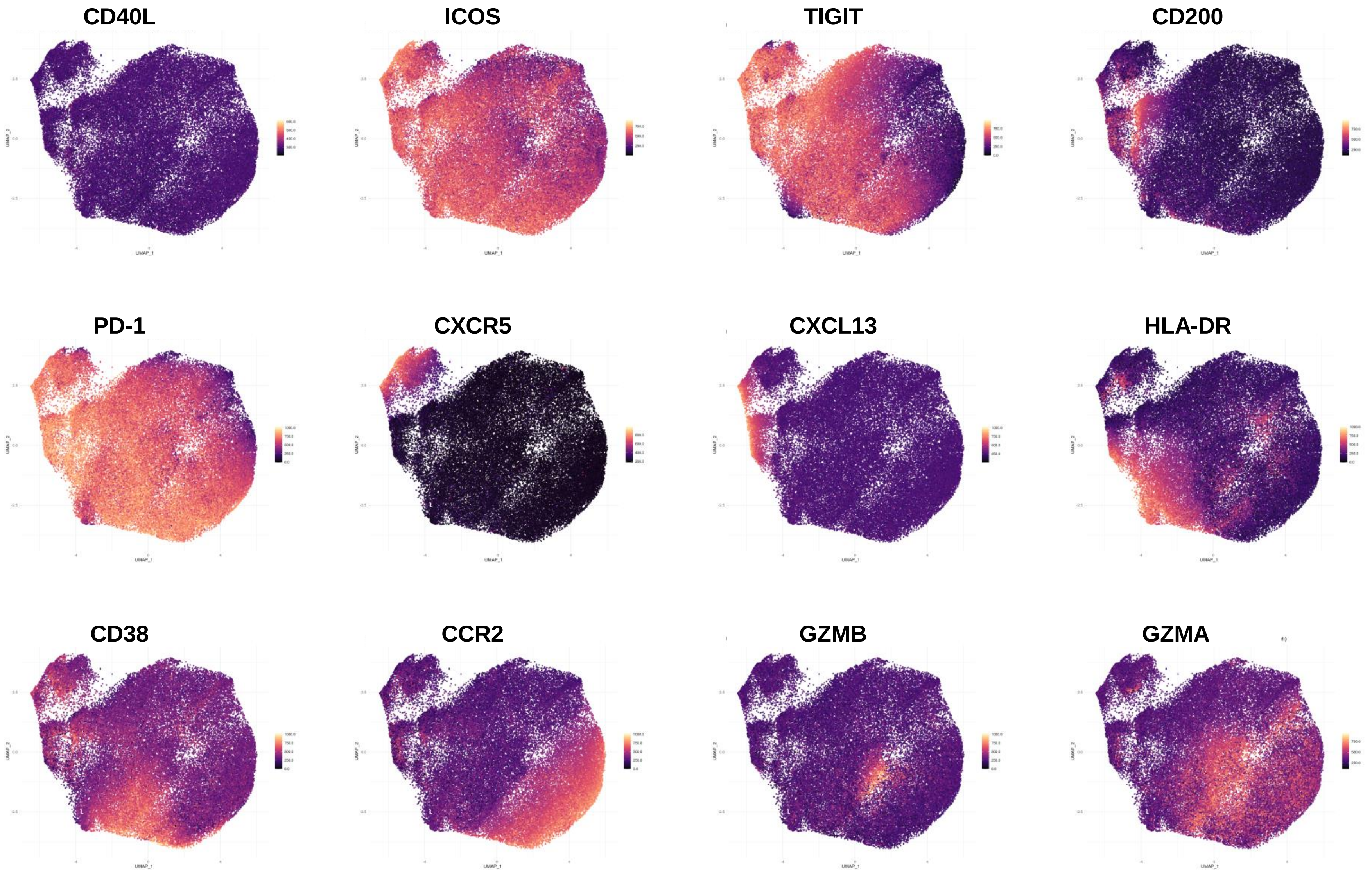

Supplementary Figure 6. Markers expression of CD4T cells in various organs of CD8TΔhPBMC mice.
Plots of each markers in uniform manifold approximation and projection (UMAP) dimensional reduction analysis.

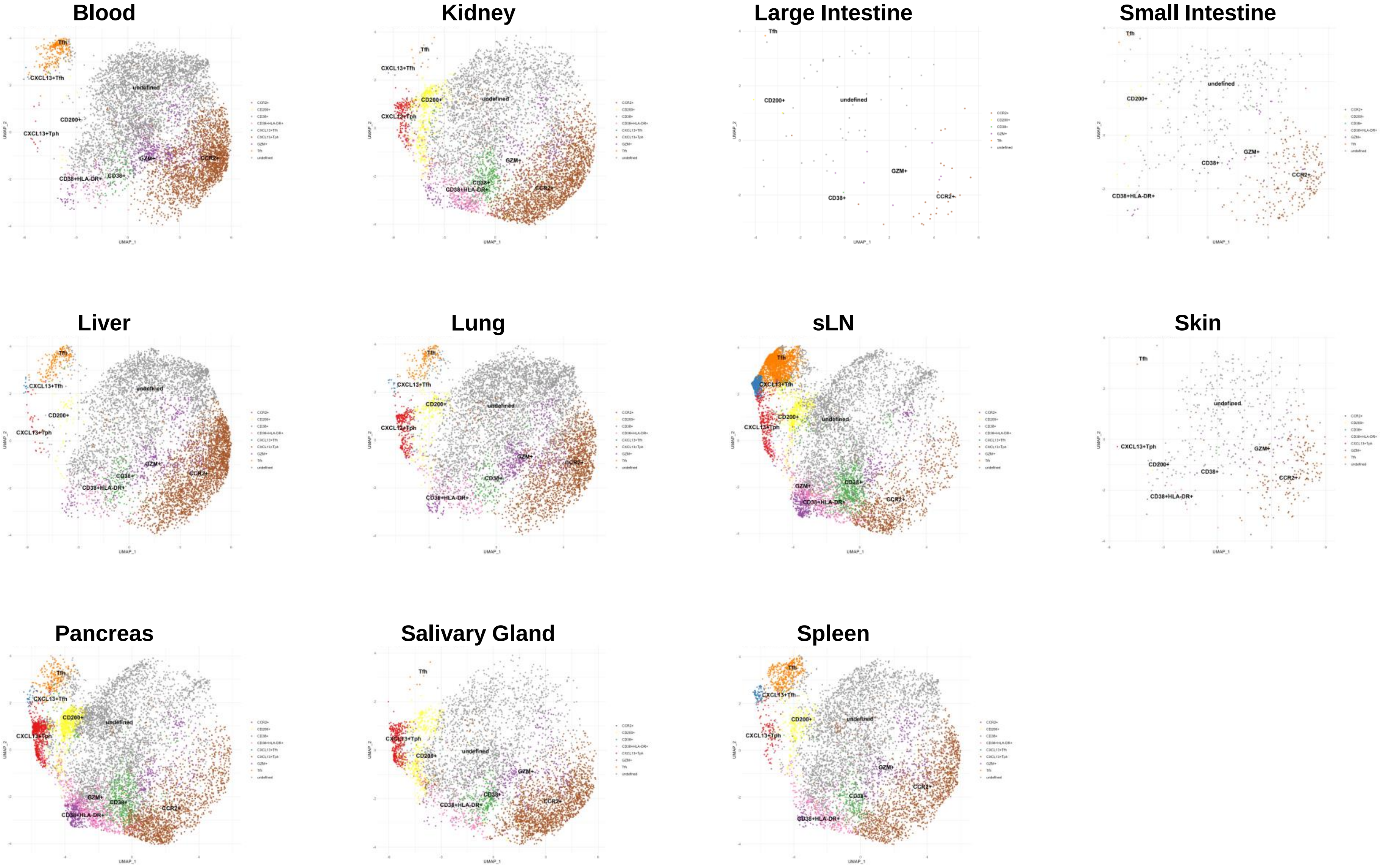

Supplementary Figure 7. Presence rate of each CD4T subset in various organs of CD8TΔhPBMC mice.
Plots of each markers in uniform manifold approximation and projection (UMAP) dimensional reduction analysis.

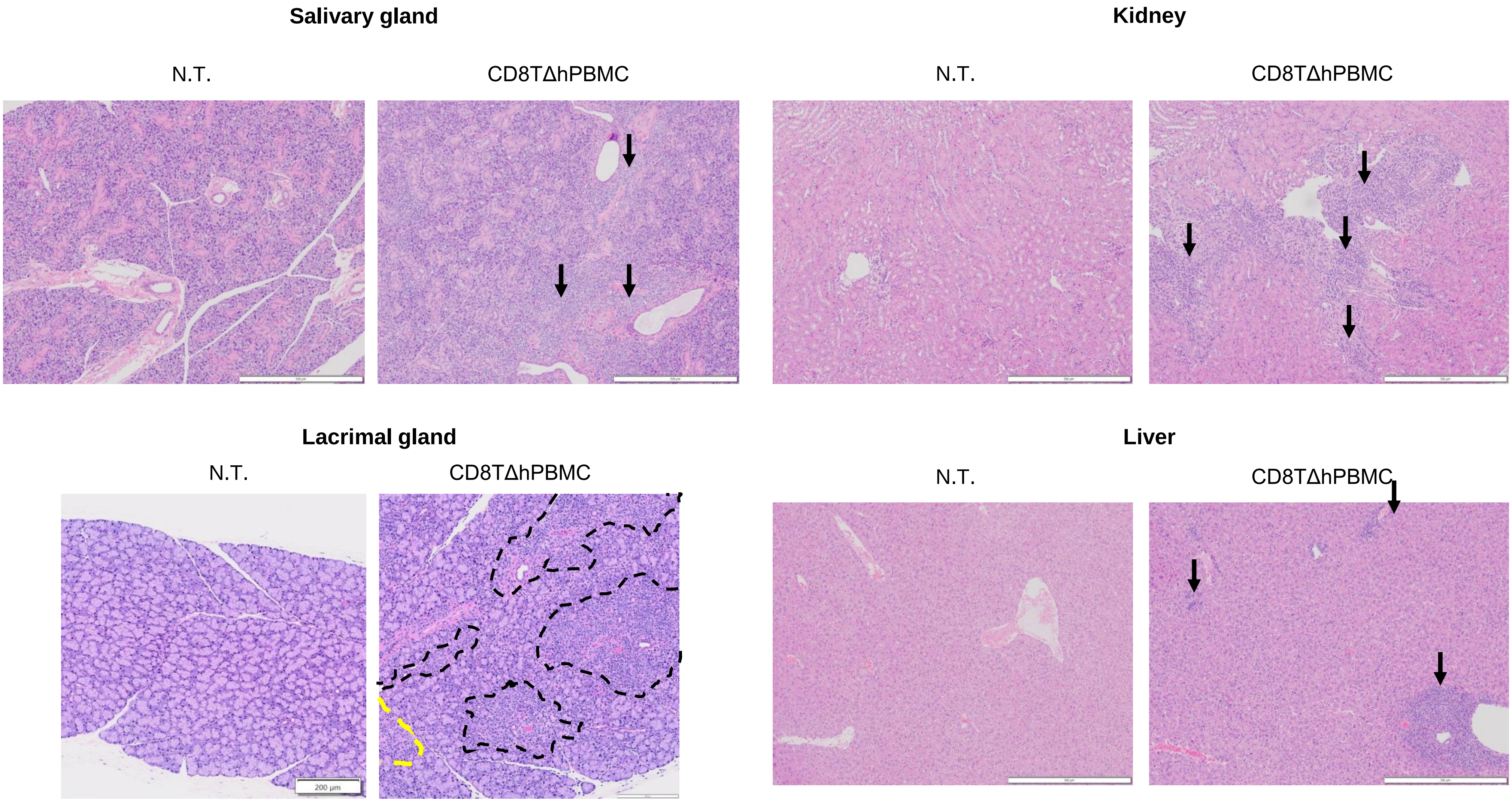

Supplementary Figure 8. Representative photomicrographs of HE stained salivary gland, lacrimal gland, kidney, liver, muscle, lung, skin and pancreas sections in CD8TΔhPBMC mice.
Black lined areas show the area of infiltrated immune cells. Scale bars, 200 μm for lacrimal gland.
Black arrows show the area of infiltrated immune cells. Scale bars, 500 μm for other tissues.

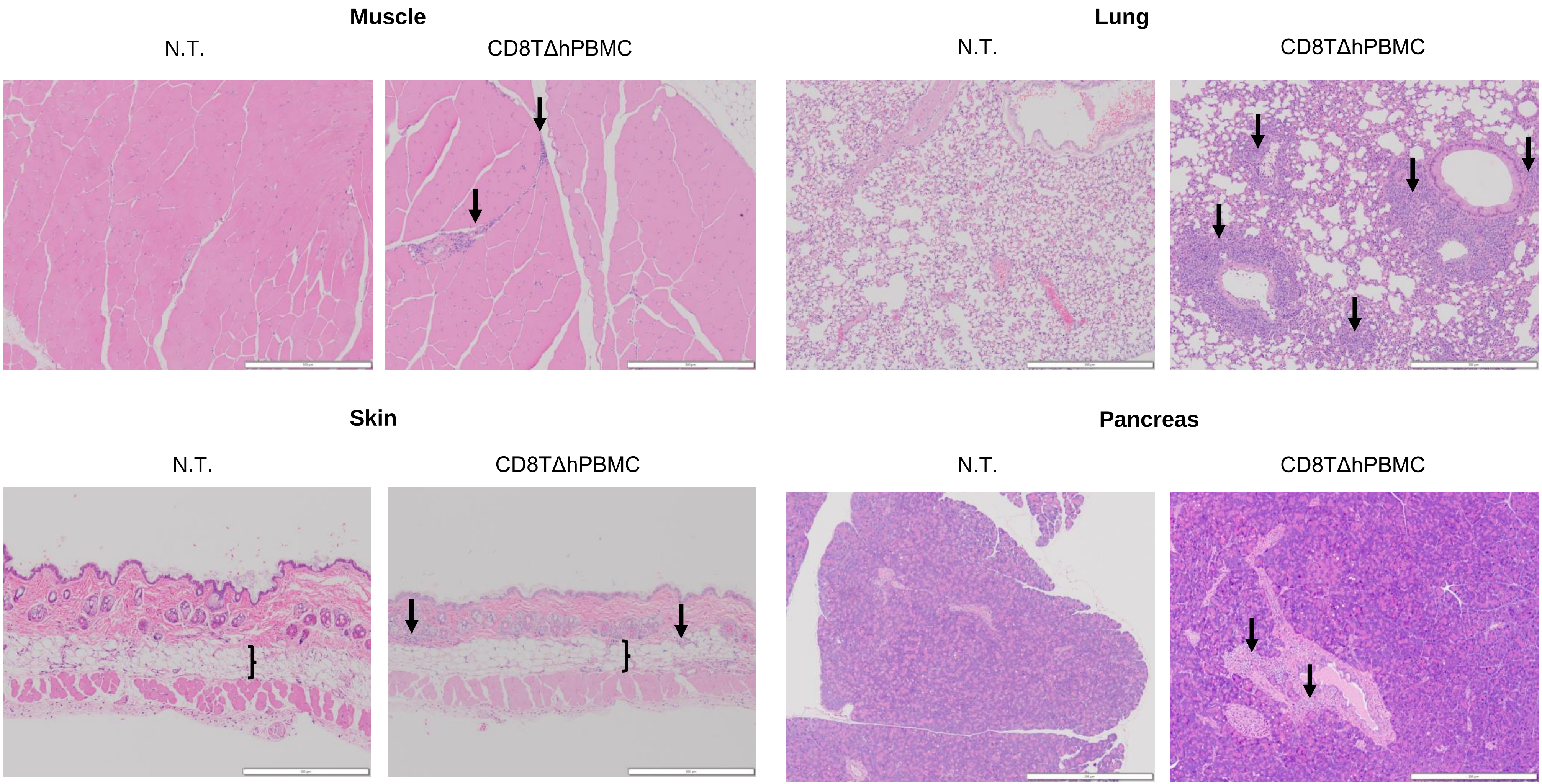

Supplementary Figure 8. Continued

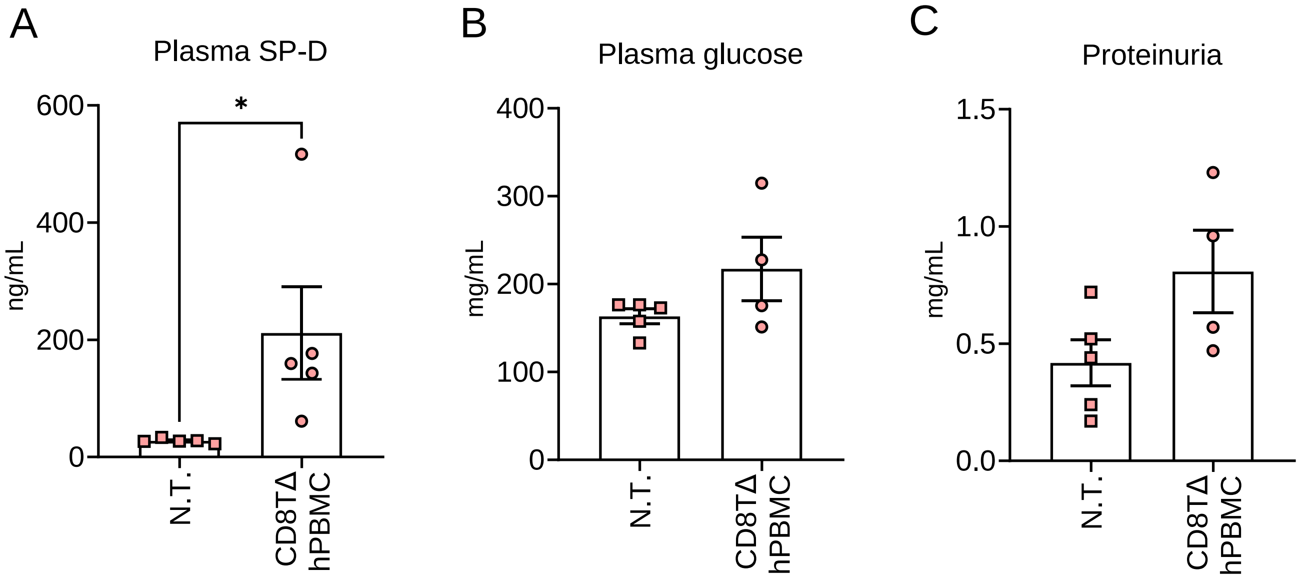

Supplementary Figure 9. Alterations in organ damage markers: plasma surfactant (SP-D), blood glucose and proteinuria in CD8TΔhPBMC mice.

NSG mice were intravenously injected with CD8T cell-depleted PBMCs. Mice were sacrificed on day 30 post-injection. (A) Plasma concentration of SP-D. (B) Plasma glucose concentration. (C) Proteinuria. Data are presented as means with SEM (n =4,5). *P < 0.05; significantly different (Student’s t-test).

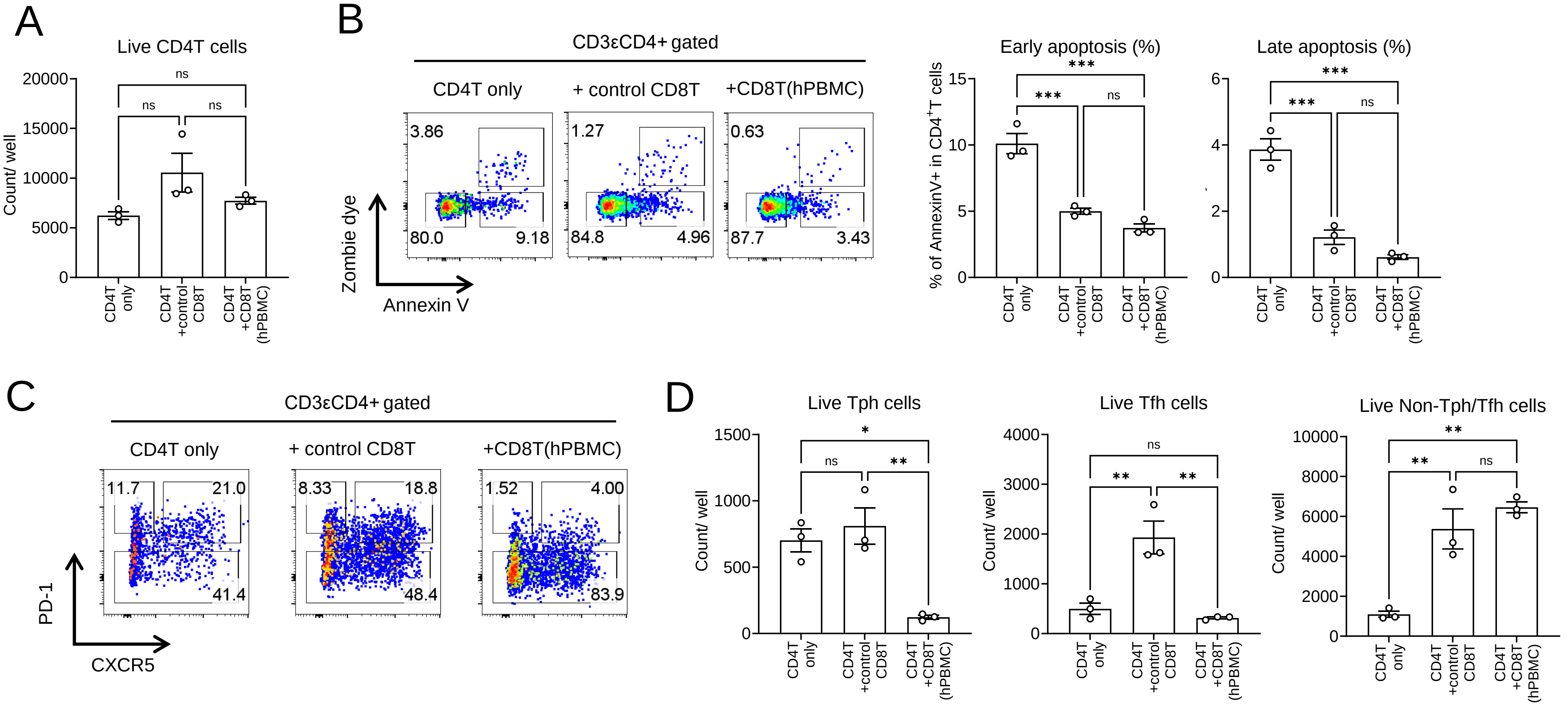

Supplementary Figure 10. CD8T cells of hPBMC mice directly inhibited Tph/Tfh cells differentiation. CD8T cells were isolated from hPBMC-transferred NSG mice. CD8T cells were cultured with CD4T cells. (A) The number of live CD4T cells. (B) Representative Annexin V/Zombie dye and CXCR5/PD-1 staining of cultured CD4T cells and the percentage of early/late apoptotic CD4T cells. (C) Representative CXCR5/PD-1 staining of cultured CD4T cells. (D) The number of live Tph, Tfh, and non-Tph/Tfh cell. Data are presented as means with SEM (n = 3). *P < 0.05, **P < 0.01, ***P < 0.001, ****P < 0.0001; significantly different (one way ANOVA with Tukey's multiple-comparison post hoc test). ns, not significant.

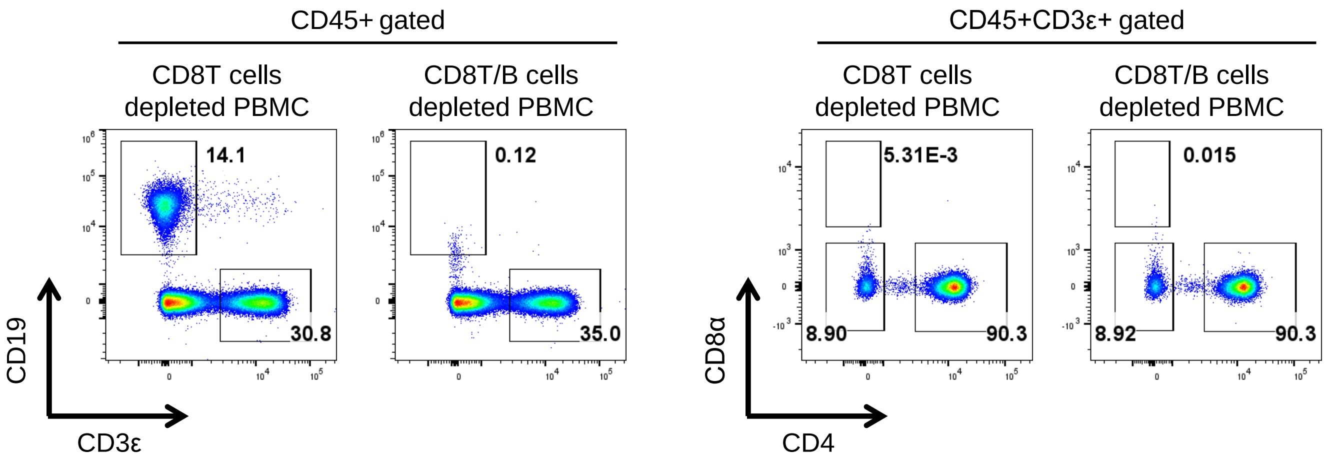

Supplementary Figure 11. Representative FACS plot of pre-transfer PBMC for CD8TΔhPBMC and CD8T/BΔhPBMC mice.

Grade	0	1	2
Weight loss	< 10%	10% to 25%	> 25%
Posture	Normal	Hunching noted only at rest	Severe hunching
Activity	Normal	Mild to moderately decreased	Stationary unless stimulated
Fur texture	Normal	Mild to moderate ruffling	Severe ruffling
Skin Integrity	Normal	Scaling of paws/tail	Obvious areas of involved skin

Supplementary Table 1. Scoring criteria for GVHD

PBMC	Age	Gende r	Race	Weight (kg)	Height (cm)	Smoke r	Blood Type	Anticoagulant	CMV Status (IgG)	HIV-1/2, Hepatitis B/C	CD3+ in CD45+ cells (%)	CD19+ in CD45+ cells (%)	CD4T in CD3+ cells (%)	CD8T+ in CD3+ cells (%)
Healthy donor 1	26	M	C	69	180	No	B+	ACDA	Negative	Negative	53.9	9.43	58.3	33.7
Healthy donor 2	28	M	HSP	80	173	No	A+	ACDA	Positive	Negative	52.4	13.3	55.4	36.4
Healthy donor 3	52	F	C	58	173	No	O+	ACDA	Negative	Negative	54.2	8.56	58.3	30.5

Supplementary Table 2. Donor characteristics of human PBMCs used in this study.

Group	Salivary gland		Lacrimal gland		Muscle		Skin	
	Immune cell infiltration	Atrophy of glandular cells	Immune cell infiltration	Atrophy of glandular cells	Immune cell infiltration	Degeneration/ necrosis of muscle fibers	Immune cell infiltration	Atrophy of subcutaneous fat
N.T.	-	-	-	-	-	-	-	-
	-	-	-	-	-	-	-	-
	-	-	-	-	-	-	-	-
	-	-	-	-	-	-	-	-
	-	-	-	-	-	-	-	-
CD8TΔhPBMC	2+	2+	2+	2+	+/-	-	+	+/-
	+	+	2+	2+	+/-	-	+/-	-
	2+	2+	2+	2+	+	-	+/-	-
	3+	3+	+/-	+/-	+	+/-	-	-
	2+	2+	+	+/-	+	-	+	-

Group	Kidney			Liver		Lung	Pancreas
	Immune cell infiltration	Perivascular fibrosis	Renal tubular degeneration	Immune cell infiltration	Degeneration/ necrosis of hepatocytes	Immune cell infiltration	Immune cell infiltration
N.T.	-	-	-	-	-	-	-
	-	-	-	-	-	-	-
	-	-	-	-	-	-	-
	-	-	-	-	-	-	-
	-	-	-	-	-	-	-
CD8TΔhPBMC	+	+/-	+/-	+/-	-	2+	+
	3+	+/-	+	+	+/-	2+	+
	+	+/-	+/-	+	+/-	2+	+/-
	3+	+	+	+	+/-	2+	-
	2+	+	+	+	+	2+	+

The qualitative score evaluated qualitatively as follows: -, No remarkable; +/-, minimal; +, slight; 2+, moderate; 3+, severe.

Supplementary Table 3. Pathological evaluation of each organ in CD8TΔhPBMC mice.

Group	Positive (n)	Negative (n)	Negative rate (%)
N.T.	0	5	100%
CD8TΔhPBMC	0	4	100%

Supplementary Table 4. Urinary glucose evaluation in CD8TΔhPBMC mice.
